# The Remodeling of Mitochondrial-Endoplasmic Reticulum Contacts by Omega-3 Fatty Acids Mitigates Dietary Advanced Glycation End Product-Driven Sertoli Cell Senescence and Oligoasthenozoospermia

**DOI:** 10.7150/ijbs.117091

**Published:** 2025-09-27

**Authors:** Zhaowanyue He, Feiyan Ge, Chuwei Li, Min Zang, Chun Cao, Jing Zhang, Shanshan Sun, Hong Zhang, Yijian Xiang, Yao Xu, Kuan Liang, Yuming Feng, Zhichuan Zou, Hui Wang, Weiqing Chen, Jie Dong, Jinzhao Ma, Shanmeizi Zhao, Li Chen, Jun Jing, Rujun Ma, Xie Ge, Bing Yao

**Affiliations:** 1 Department of Reproductive Medicine, Jinling Hospital, Affiliated Hospital of Medical School, Nanjing University, Nanjing, Jiangsu, China, 210002.; 2 State Key Laboratory of Reproductive Medicine and offspring Health, Nanjing Medical University, Nanjing, Jiangsu, China, 211166.; 3 The First School of Clinical Medicine, Southern Medical University, Guangzhou, China, 510515.; 4 Department of Reproductive Medicine, Affiliated Jinling Hospital, Nanjing Medical University, Nanjing, China, 210002.; 5 Jinling Clinical Medical College, Nanjing University of Chinese Medicine, Nanjing, China, 210002.; 6 State Key Laboratory of Analytical Chemistry for Life Science, Nanjing, China, 210023.

**Keywords:** Male infertility, Advanced glycation end products, Sertoli cell, Mitochondria‒endoplasmic reticulum contacts, Omega-3 fatty acids, Receptor for AGE

## Abstract

Dietary components or patterns have been shown to affect male fertility. The increasing intake of processed foods rich in advanced glycation end products (AGEs) may threaten spermatogenesis. However, the key cell type affected by AGEs in spermatogenic microenvironment remains unspecified. Furthermore, given that subcellular organelle interactions, particularly communications between mitochondria and endoplasmic reticulum (ER), are of paramount importance in male fertility, it is worthwhile to investigate dynamic changes of mitochondria-ER contacts (MERCs) in AGE-driven spermatogenesis dysfunction. In this study, we found that serum AGEs levels increased in patients with oligoasthenozoospermia (OAZ), which was accompanied by decreased inhibin B levels, leading us to explore the effect of AGEs on Sertoli cells. *In vivo* experiments revealed that AGEs-rich diet disrupted spermatogenesis and induced Sertoli cell senescence and dysfunction in mice. We further confirmed that AGEs elicited an increase in MERCs, as well as ER stress and mitochondrial dysfunction in Sertoli cells. Omega-3 polyunsaturated fatty acids (omega-3), which are a category of dietary supplements with the potential to improve male fertility, were employed in the rescue experiment. We demonstrated that omega-3 mitigate dietary AGE-induced Sertoli cell senescence and OAZ via the remodeling of MERCs, highlighting the AGE-RAGE axis as a potential target for treating male infertility.

## Introduction

Approximately 8-12% of couples are affected by infertility worldwide, with male factors accounting for approximately half of these cases 1, 2. Over the past few decades, there has been a continuous decrease in male fertility parameters worldwide. Male infertility can stem from a wide range of causes, which may include congenital conditions, acquired disorders, or idiopathic factors that impact spermatogenesis. In addition, diet and nutritional status are among the significant determinants of male fertility 3, 4. Increasing evidence suggests that dietary patterns are closely associated with semen quality and can influence reproductive outcomes 5. Metabolic factors are often relevant to decreased sperm concentration and motility, which are typical characteristics of oligoasthenozoospermia (OAZ) 6. OAZ is a common cause of male infertility, accounting for 30%-40% of male infertility cases. Given this, it is of great clinical significance to delve into the impact of diet and nutritional factors on male fertility and their underlying mechanisms.

As society continues to advance, the consumption of processed foods, including high levels of processed meats, sugar-laden beverages, fried foods, and high-fructose items, has become increasingly prevalent 7, 8. The Maillard reaction is a nonenzymatic interaction that occurs during the production and processing of these foods and results in the formation of advanced glycation end products (AGEs) 9, 10. When ingested, 10-30% of dietary AGEs are absorbed into systemic circulation as small molecules. While short-term intake does not significantly affect body AGE levels, long-term consumption raises both free and bound AGEs due to accumulation and conversion 11. Free AGEs, with molecular weights <5 kDa, are rapidly absorbed via simple diffusion, while bound AGEs are hydrolyzed in intestinal cells and can enter circulation 12. Once absorbed, AGEs bind to Receptor for AGE (RAGE) on cell membranes, and activate RAGE-dependent signaling pathways 12, 13. AGEs accumulate during normal aging and in the context of age-related diseases, and AGEs trigger pathways that accelerate cellular senescence 14. Pathological aging, caused by AGEs, is associated with a range of conditions including autoimmune and inflammatory diseases, neurodegenerative disorders, diabetes/diabetic nephropathy, cardiovascular diseases, and bone degenerative diseases 11. Several clinical studies have indicated potential adverse effects of AGEs on male fertility 15-17. However, the mechanism underlying AGE-induced spermatogenic dysfunction remains to be explored, and such studies may provide targets for treating spermatogenic dysfunction.

Spermatogenesis strongly relies on the testicular microenvironment. Sertoli cells are essential somatic cells within the seminiferous tubules, and they play a key role in sustaining the microenvironment that is needed for germ cell development 18. The major functions of Sertoli cells include forming the blood‒testis barrier (BTB) 19, providing structural and nutritional support to germ cells 20, and secreting cytokines to regulate spermatogenesis 21, 22. Consequently, impairments in Sertoli cell functions may lead to the disruption of spermatogenesis 23-25. Abnormalities in Sertoli cells are considered indicators of testicular aging 26, 27. Since the accumulation of AGEs in various organs is a hallmark of aging, investigating the impact of AGEs on Sertoli cell functions will significantly enhance our understanding of testicular senescence mechanisms.

Studies have shown that Sertoli cells that are exposed to metabolic stress trigger specific adaptive responses. Dysregulation of cellular organelles, particularly the endoplasmic reticulum (ER) and mitochondria, significantly influences stress-induced pathophysiological changes 28. Mitochondria‒ER contact sites (MERCs) function as transient signaling platforms that regulate vital cellular processes by promoting the exchange of substrates such as calcium across the ER and mitochondria. MERCs regulate the transfer of calcium ions (Ca²⁺) between the ER and mitochondria via the inositol 1,4,5-trisphosphate receptor (IP3R), and allows Ca²⁺ to passively enter the outer mitochondrial membrane (OMM) through voltage-dependent anion channels (VDAC1). Notably, dysfunctions in MERCs are closely related to various diseases 29-31. Also, intracellular calcium accumulation due to MERCs abnormality is indicative of cellular aging. It has been demonstrated that MERCs have a significant impact on the aging of skeletal muscle cells, cardiomyocytes, and intestinal stem cells, as well as therapy-induced senescence 32-35. As previously reported, AGEs caused imbalance in the intracellular redox state and may damage cellular homeostasis through MERCs 36. Therefore, changes in MERCs induced by AGEs may affect the function of Sertoli cells by impacting the ER and mitochondria, disrupting calcium homeostasis, and inducing senescence, thereby affecting their functions.

Polyunsaturated fatty acids (PUFAs), which are predominantly derived from fish, algae, and phytoplankton, are dietary factors that are essential for human health. Omega-3 polyunsaturated fatty acids (omega-3), a key subclass of PUFAs with antioxidant properties, have shown significant clinical value in treating metabolic diseases, including OAZ caused by metabolic disorders 37, 38. Both ER and mitochondria are membrane-bound organelles, characterized by their lipid-rich membranes. Communication between these organelles is facilitated by specialized membrane regions known as mitochondria-associated membranes (MAMs) 39. By modulating the lipid composition of cell membranes, omega-3 increase membrane fluidity, and they may play a role in restoring normal communication between the ER and mitochondria 40-42. Analysis of the protective effects of omega-3 against AGE-related OAZ, as well as the regulatory mechanisms involved, may reveal candidate treatments for male subfertility associated with metabolic disorders.

In this study, we detected increased levels of AGEs in the sera of patients with OAZ. Our *in vivo* experiments revealed that a diet rich in AGEs disrupted spermatogenesis in mice by triggering Sertoli cell senescence and functional disruption. We further confirmed that AGEs induced an increase in MERC numbers, ER stress and mitochondrial dysfunction in Sertoli cells. Additionally, we confirmed that AGEs induce these pathological changes by binding to the RAGE. Notably, our study demonstrated that omega-3 treatment alleviated the detrimental effects of AGEs on Sertoli cells and ameliorated spermatogenesis. These results suggest that AGEs contribute to OAZ development and that omega-3 play a protective role, suggesting a new perspective for the treatment of male infertility in the clinic.

## Materials and Methods

### Analysis of serum and sperm quality in patients

24 patients with OAZ and 24 healthy controls were recruited from the clinical laboratory of Nanjing Jinling Hospital during routine semen analysis from September 2023 to January 2024. The specimens were collected between 8:00 and 11:00 a.m. The participants were aged 25 to 44 years. Blood samples were centrifuged for 10 minutes at 2,000 × g at 4°C, after which serum samples were extracted and stored at -70°C. The sperm quality was assessed using the WLJY-9000 computer-assisted system. According to the sixth edition of the WHO Laboratory Manual for the Examination and Processing of Human Semen, OAZ is characterized by oligospermia (<15 × 10^6^ spermatozoa/mL) and/or asthenozoospermia (<32 % progressive motility).

### Animals and experimental design

**Figure 1:**
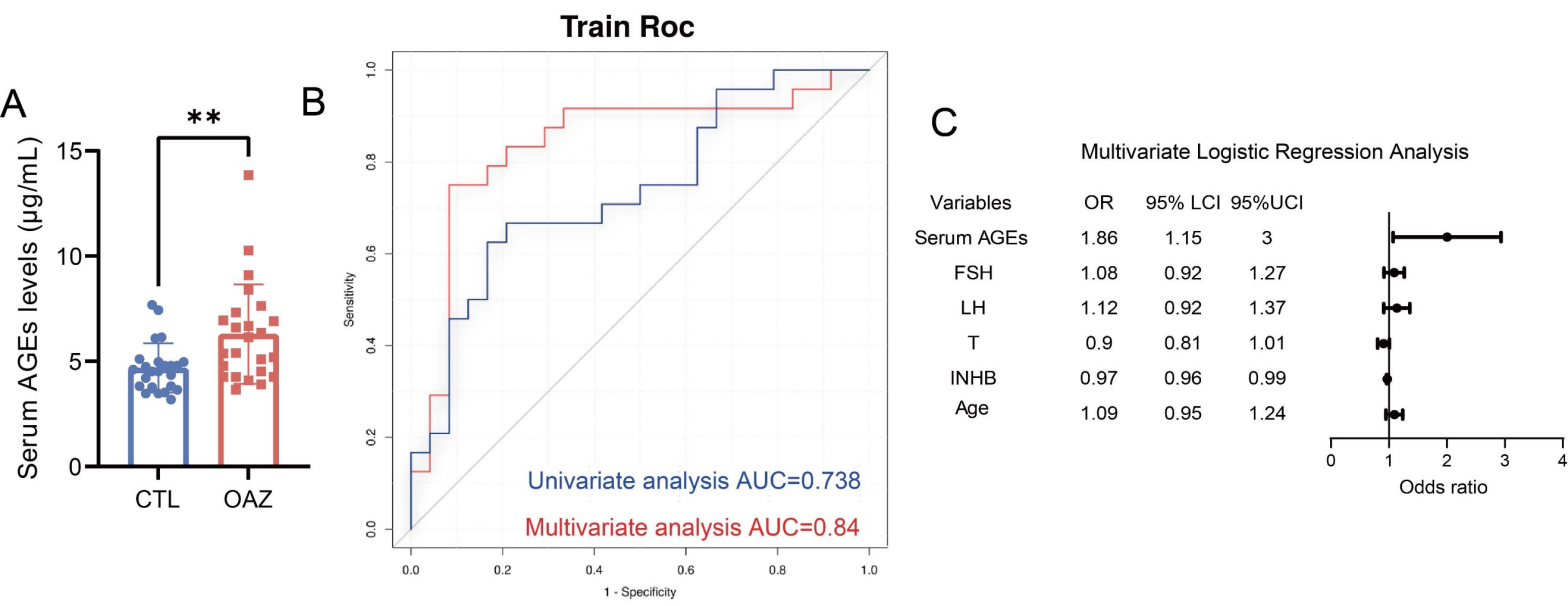
** Serum AGEs levels are significantly higher in patients with OAZ.** (A) Serum AGEs levels in CTL and OAZ patients were analyzed by ELISA. (B-C) Univariate Logistic regression analysis (blue lines) was performed to determine whether serum AGEs is a risk factor for OAZ occurrence. Multivariate Logistic regression analysis (red lines) assessed whether serum AGEs, FSH, LH, T, INHB, and age are OAZ risk factors. A ROC curve was plotted with an AUC calculated. The forest plot from the Multivariate Logistic regression analysis showed the Odds Ratios (OR) and 95% Confidence Intervals (CI) for each factor. An unpaired t-test was used for two - group comparisons. A two - sided p - value < 0.05 was statistically significant, with p < 0.01 defined as highly significant (**).

Thirty male ICR mice were obtained from the Animal Core Facility of Nanjing Medical University (Nanjing, China). The ICR mice were housed in an environment with a 12-h light/12-h dark cycle, a temperature of 20-26°C, and constant humidity (70%). Food and water were provided ad libitum. The mice were randomly assigned to three distinct groups after the acclimatization period. Each group included 10 animals and was subjected to the indicated dietary interventions for a continuous period of 16 weeks (Fig. 1A). The groups included (1) a control group (CTL) that received a diet low in AGEs (AIN-93G unbaked), (2) an AGEs-rich diet group (dAGEs) (AIN-93G baked for 1 h at 160°C) as described in previous studies 43, and (3) an dAGEs group gavaged with omega-3 PUFAs (OMEGA 3 TREASURE, Shanghai, China) at 2 g/kg body weight every 2 days for the last 4 weeks (dAGEs+Omega-3). CTL and dAGEs groups received an equal volume of corn oil as a negative control via intragastric administration.

The experimental protocols were approved by the Ethics Committee of Nanjing Jinling Hospital and complied with the National Laboratory Animal Care and Use Research Committee Guidelines. The mice were euthanized, and the blood samples were collected in enzyme-free sodium centrifuge tubes after acquiring via orbital sinus puncture, followed by centrifugation to separate sera. Subsequent cervical dislocation was performed before collecting testis and epididymis samples.

### Analysis of mouse sperm quality

The assessment of sperm quality in mice was conducted using methods previously detailed 20, 42. The human tubal fluid medium was pre-warmed at 37°C. Fresh cauda epididymides were carefully transferred into 0.4 mL of the pre-warmed medium and finely minced using a sterile needle. After a 5-minute incubation at 37°C, the sperms were gently resuspended, and a 10 μL aliquot was transferred to a hemocytometer for examination and counting under a microscope.

### Enzyme-linked immunosorbent assay (ELISA)

The content of AGEs in human sera was measured using Human AGE ELISA Kit (ABclonal, Shanghai, China), and the content of AGEs in mouse testis homogenate or sera was measured using AGE ELISA Kit (Biorbyt, Cambridge, UK), according to the manufacturer's instructions. The AGE concentrations in the samples were then expressed as µg/µL serum or µg/mg protein. To detect cytokines in testes, tissues were obtained and ground into homogenate. The supernatants were taken for ELISA tests (ABclonal, Shanghai, China) according to the protocols.

### Electron microscopy

Small fragments of testicular tissue were collected and fixed in 2.5% glutaraldehyde. The samples were sequentially stained with 1% osmium tetroxide, 2% uranyl acetate, and lead citrate. The cellular observation was conducted utilizing a Hitachi transmission electron microscope. The minimal proximity between mitochondria and the ER was assessed by quantifying the shortest inter-organellar distance between these two structures.

### Analysis of BTB integrity

The integrity of the BTB was evaluated as previously described 25. Mice were randomly selected from each group and anesthetized with pentobarbital sodium (5 mL/kg). The testes were surgically exposed, and 20 µL of freshly prepared EZ-Link Sulfo-NHS-LC-Biotin (Thermo Fisher, MA, USA) was administered via multipoint intratesticular injections into the interstitial space. Following a 30-minute diffusion period, the mice were euthanized, and the testes were dissected out and immediately snap-frozen in liquid nitrogen. Cryosections of testicular tissue were prepared at 10-µm thickness.

Sections were fixed with 4% formaldehyde for 10 minutes and subsequently incubated with Alexa Fluor 488-conjugated streptavidin (Invitrogen, CA, USA) for 1 hour at room temperature. Images of seminiferous tubules were acquired using a fluorescence microscope. For quantitative analysis, 50-60 circular or elliptical cross-sections of seminiferous tubules were randomly selected per group. The ratio of biotin diffusion distance (D_biotin_) to tubule radius (D_radius_) was calculated. For elliptical tubules, D_radius_ was defined as the average of the major and minor axes.

### Isolation and culture of primary mouse cells

Primary mouse Sertoli cells were isolated via a two-step enzymatic digestion protocol, as previously outlined 44. The isolated cells were maintained in DMEM/F12 medium supplemented with 10% fetal bovine serum (Gibco, CA USA) at 34°C under 5% CO_2_. To eliminate germ cells, the cells were treated with 20 mM Tris for 3 minutes, followed by washing with PBS and further culturing until they were ready for use.

Rosa26-mTmG mice were obtained to isolate primary germ cells, using differential adhesion method based on a previously published protocol 45. The isolated germ cells were then added to pre-separated and cultured primary Sertoli cells. The Sertoli cells were initially cultured for 3 days, after which germ cells were introduced at a ratio of 1:5 to facilitate the formation of ectoplasmic specializations. On the 5^th^ day of co-culture, images were captured using a fluorescence microscope, and 5 random fields of view were selected. Then the number of germ cells that had adhered was quantified, and the percentage reduction of their number was calculated.

### Cell culture and treatment

TM4 mouse Sertoli cells were cultured in DMEM/F12 medium supplemented with 10% fetal bovine serum at 37°C under a 5% CO_2_ atmosphere. When approaching confluence, cells were detached via trypsinization and seeded at an appropriate density. After 1 day of culture, experimental agents were introduced once the cells reached 70% confluence. Cells were treated with control bovine serum albumin (BSA) (200 µg/mL) or AGEs-modified BSA (AGEs-BSA, abcam, Cambridge, UK, 200 µg/mL). For rescuing experiments, omega-3 (200 μM) or FPS-ZM1 (10 μM) were used together with AGEs-BSA. The doses of these reagents used for cell treatment were determined based on previously published studies 20, 46. Generally, cells are collected after 24 hours of treatment.

### Analysis of apoptosis

Apoptosis was evaluated using the Annexin V-FITC/Propidium Iodide (PI) Staining Kit (Keygen, Shanghai, China). TM4 cells were collected by trypsinization without EDTA, centrifuged, and washed twice with PBS. After resuspending in 500 μL of binding buffer, the cells were stained with 5 μL each of FITC and PI, gently mixed, and incubated at room temperature in the dark for 15 minutes. Apoptosis was analyzed by flow cytometry using FITC and PI channels to calculate the percentage of apoptotic cells. Data analysis was performed using Flowjo10 software.

### Transepithelial electrical resistance (TER) measurements

To evaluate cellular barrier functions* in vitro*, the TER was measured using a Milli-cell ERS System (Merck Millipore, MA, USA) every day. Three different areas of the units was measured, and the TER value was calculated using the following formula:

TER (Ω·cm^2^) = (treatment resistance (Ω)-background resistance (Ω)) × membrane area (cm^2^)

### Proximity Ligation Assay (PLA)

The PLA was performed using the NavinciFlex^TM^ Cell MR In Situ Detection Kit (Navinci, Uppsala, Sweden). All the incubation steps were performed in a humid chamber. TM4 cells were subjected to treatment and fixed with 4% paraformaldehyde for 20 minutes. To allow antibodies and other reagents to enter the cells, TM4 cells were treated with 0.5% Triton X-100 for 15 minutes to enhance cell membrane permeability. The slides were washed using PBS (pH 7.4) on a shaking platform three times, blocked with blocking solution at 37 ºC for 1 hour, and then incubated with diluted primary antibodies against two different proteins overnight at 4ºC. Subsequently, the samples were incubated with PLA probes that matched the primary antibodies diluted in Navenibody M1 and Navenibody R2 in Navenibody Diluent at 37ºC for 1 hour. Following another wash, the samples were subjected to DNA ligation with enzyme A in buffer A for 30 minutes and enzyme B in buffer B for 1 hour, both at 37ºC. Finally, the samples were stained with DAPI. Keep the slides moist throughout the incubation. As a negative control, samples were incubated with only one primary antibody. The PLA signals, which reflect the interaction of these two different proteins, appeared as distinct fluorescent spots and were analyzed using confocal microscopy. PLA puncta levels were quantified on the basis of the acquired images.

### Protein aggregation assay

Protein aggregation was evaluated with the PROTEOSTAT^®^ Protein Aggregation Kit (Enzo Life Sciences, Raamsdonksveer, Netherlands). The lysates of TM4 cells were sonicated on ice to extract the proteins. After centrifugation at 12,000 rpm for 10 minutes at 4°C, the supernatants were carefully collected. Protein concentrations were determined with a BCA Protein Assay Kit (Thermo Fisher, MA, USA), and equal amounts of protein were mixed thoroughly with ProteoStat dye and incubated at room temperature for 15 minutes, allowing the dye to bind specifically to exposed hydrophobic regions of aggregated proteins. The intensity of protein aggregation was then quantified in term of fluorescent signal intensities using a microplate reader (Bio-Rad, USA).

### Detection of reactive oxygen species (ROS) in mouse testicular cryosections

Freshly isolated mouse testes were snap-frozen in liquid nitrogen. The tissues were embedded in optimal cutting temperature (OCT) compound, sectioned at a thickness of 10 µm using a cryostat, and maintained at -20°C. Then, the sections were collected on positively charged glass slides. After drawing circles using a histochemical pen around the tissues to prevent the leakage of antibodies, the ROS staining solution (Servicebio, Wuhan, China) was applied within the circles and the slides were incubated at 37°C in the dark for 30 minutes.

The slides underwent a series of three washes in PBS (pH 7.4). Each wash was carried out on a rocking shaker and lasted for 5 minutes. Then the sections were incubated with DAPI staining solution (Beyotime, Shanghai, China) for 10 minutes at room temperature in the dark. Following washing three times with PBS (pH 7.4) for 5 minutes on a rocking shaker, the sections were briefly dried, and mounted with antifade mounting medium. Fluorescence images were acquired on a fluorescence microscope, with ROS-positive signals (green fluorescence) and DAPI-stained nuclei (blue fluorescence) captured separately and merged.

### TUNEL staining of mouse testicular paraffin sections

The Fluorescein (FITC) TUNEL Cell Apoptosis Detection Kit (Servicebio, Wuhan, China) was used to detect apoptotic cells in testicular paraffin sections according to the manufacturer's instructions. Testicular paraffin sections were de-waxed and gently rinsed with PBS. Sections were treated with 100 µL of Proteinase K working solution at 37°C for 20 minutes, followed by three 5-minute PBS washes. Sections were then incubated with 50 µL of equilibration buffer at room temperature for 10 minutes. Freshly prepared TdT incubation buffer (57 µL) was applied to each section and incubated at 37°C for 1 hour in the dark. After washing with PBS, sections were stained with DAPI solution for 8 minutes at room temperature in the dark. Fluorescence images were captured using a fluorescence microscope. Apoptotic nuclei were identified by FITC green fluorescence.

### Statistical analysis

Clinical data were processed using SPSS 23.0. Univariate logistic regression and multivariate logistic regression analyses were conducted to assess the independent associations between the level of serum AGEs and the indicators of OAZ. The discriminative ability of the model was evaluated using ROC curve analysis. The AUC was used to measure the diagnostic accuracy of the model. The graphs were plotted using GraphPad Prism 9.5, and data were presented as the means ± standard deviations (SD). Data were assumed normally distributed if Shapiro-Wilk test p>0.05 and homoscedastic if Levene's test p>0.05. For independent samples meeting both normality and homoscedasticity, two-group comparisons were analyzed utilizing the independent samples t-test, while multi-group comparisons were analyzed employing one-way ANOVA followed by Bonferroni-adjusted post hoc tests. When normality held but variances were unequal (heteroscedasticity), Welch's t-test (two groups) or Welch's ANOVA with Games-Howell post hoc tests (≥ 3 groups) were applied. Non-normally distributed data were analyzed via Mann-Whitney U test (two groups) or Kruskal-Wallis test with Dunn-Bonferroni correction (≥ 3 groups). All statistical tests were conducted at a two-sided significance level of 0.05. The significance levels were indicated by* p* < 0.05 (*), *p* < 0.01 (**), *p* < 0.005 (***), and *p* < 0.001 (****).

## Results

### Serum AGEs levels are significantly higher in patients with OAZ

To explore the potential link between AGEs levels and OAZ, sera samples from 24 patients with OAZ and 24 healthy controls (CTL) were collected, AGEs levels were measured (Table S1, Table S2). The results revealed that the serum AGEs levels in the OAZ group were significantly greater than those in the CTL group (Fig. 1A), indicating a relationship between excessive AGEs levels and disrupted spermatogenesis.

We further performed univariate and multivariate logistic regression analyses (Table S3) and found that a high level of serum AGEs was a risk factor for OAZ (*p*=0.011). The area under the curve (AUC) for the prediction of OAZ on the basis of serum AGEs levels was 0.738 [95% CI (0.595, 0.881)] (Fig. 1B), which holds certain diagnostic value. The predictors included in the multivariate logistic regression analysis were serum AGEs levels (OR 1.86, 95% CI 1.15-3.00), follicle-stimulating hormone (FSH) levels (OR 1.08, 95% CI 0.92-1.27), luteinizing hormone (LH) levels (OR 1.12, 95% CI 0.92-1.37), testosterone (T) levels (OR 0.90, 95% CI 0.81-1.01), inhibin B (INHB) levels (OR 0.97, 95% CI 0.96-0.99), and age (OR 1.09, 95% CI 0.95-1.24) (Table S3). These predictors showed stronger predictive power for the development of OAZ, with an AUC of 0.824 [95% CI (0.69-0.958)] (Fig. 1C). The forest plot indicated that among these indicators, serum AGEs levels had a more significant impact. On the basis of these findings, we propose that elevated AGEs levels may contribute to the development of OAZ.

### AGEs disrupt spermatogenesis and induce testicular senescence, and omega-3 treatment mitigates these effects

**Figure 2:**
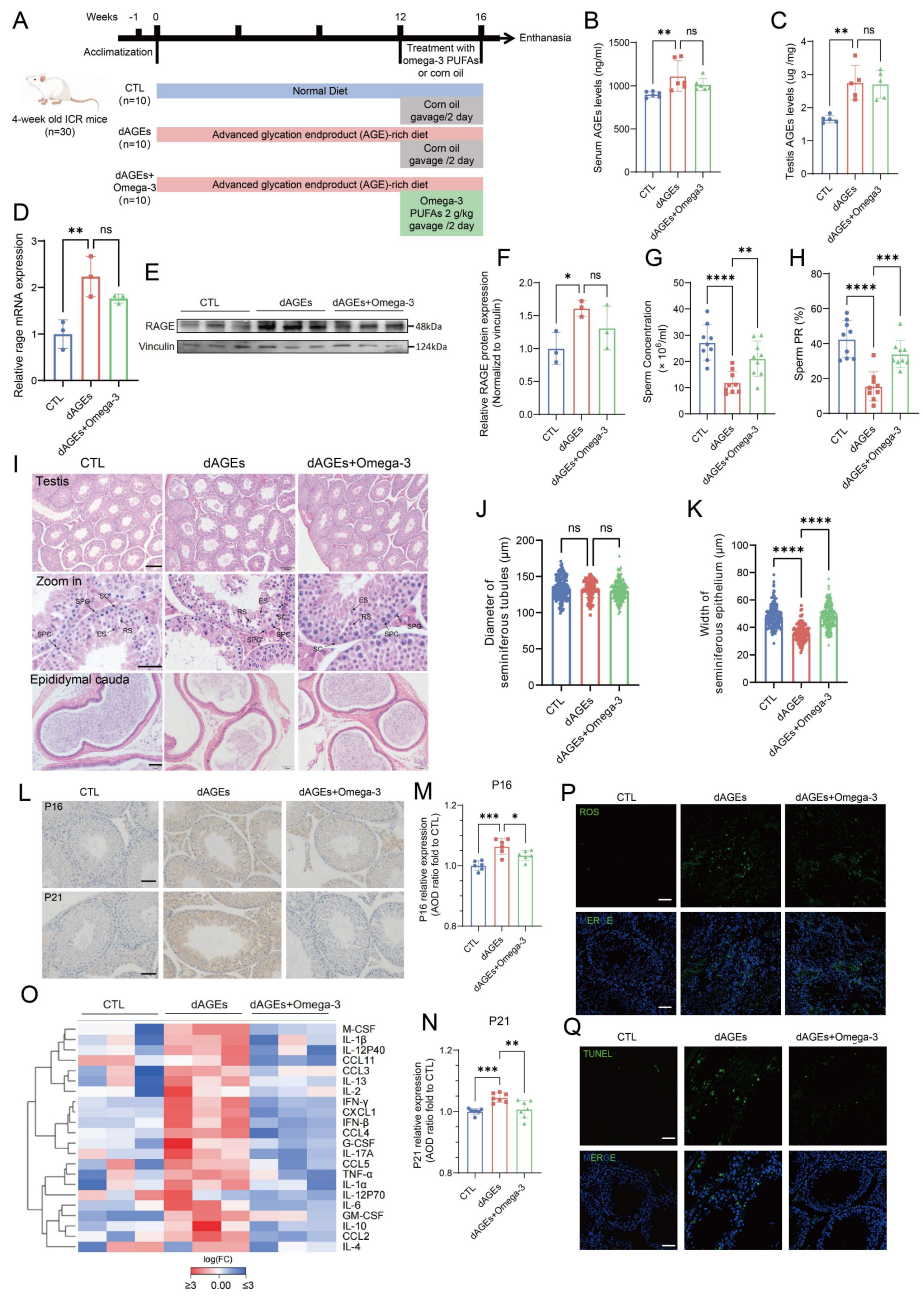
** Omega-3 mitigates AGEs effects on spermatogenesis and testicular senescence.** (A) Animal Experimental Design: All animals were randomly divided into three groups: a control group (CTL), an advanced glycation end product-rich diet group (dAGEs) for 16 weeks, and a group fed an AGEs-rich diet supplemented with omega-3 PUFAs for the last 4 weeks (dAGEs + Omega-3). (B, C) Concentrations of AGE in sera(B) and testis(C) were analyzed by ELISA. (D-F) The mRNA levels (D) and the protein expression(E) of RAGE in mice testis. Protein expression was quantified by ImageJ and normalized (F). (G, H) Sperm concentration (G) and sperm progressive motility (PR) (H) of the mice after 16 weeks of treatment. (I) Representative photographs of testicular (upper and middle) and epididymal tail (lower) morphology with HE staining. Scale bars: for testis pictures, 100 μm for the original picture and 25 μm for the enlarged picture; for epididymal tail picture, 50 μm. Spermatogonia (SPG), Spermatocytes (SPC), Round Spermatids (RS), Elongated Spermatids (ES) and Sertoli Cells (SC) are indicated. (J-K) Diameter assessment and epithelial thickness quantification of each seminiferous tubule across different groups. (L-N) The expression of P16 (upper) and P21 (lower) proteins in mouse spermatogenic tubules was assessed by immunohistochemistry. Scale bars: 50 μm (M, N) Quantitative analysis using Average Optical Density (AOD) to assess the expression levels of proteins in tissue sections and normalized. (O) The levels of SASP factors in mouse testes were displayed using a heatmap. (P, Q) Representative images of ROS staining (P) and TUNEL staining (Q) in mice testicular spermatogenic tubules. Scale bars: 50 μm. Statistical analysis between multiple groups was performed by one-way ANOVA. A two-sided p-value < 0.05 was considered to be statistically significant. The level of significance defined as p < 0.05 (*), p < 0.01 (**), p < 0.005(***), p < 0.001 (****).

To investigate the effects of dAGEs intake on spermatogenesis, we fed mice an AGEs-rich diet for 16 weeks. Moreover, to propose a safe and effective clinical treatment strategy, we administered omega-3 via intragastric gavage during the last 4 weeks (dAGEs + omega-3) (Fig. 2A). The ELISA results revealed that mice in the dAGEs group exhibited significant increases in both sera and testicular AGEs levels. However, omega-3 did not significantly affect AGEs levels (Fig. 2B-C). RAGE is a pivotal cell surface Receptor for AGE that activates intracellular signaling pathways upon the binding of AGEs 42. Both the mRNA and protein levels of RAGE were increased in the testes of the dAGEs group (Fig. 2D-F). These results suggest that AGEs directly target cells within the testicular microenvironment.

Both the sperm concentration and motility were substantially decreased in the dAGEs group (Fig. 2G-H). Although there was no significant change in body weight, the testis and epididymis indices were markedly decreased (Fig. S1A-C). Compared with mice in the dAGEs group, mice in the omega-3-treated group exhibited significantly greater sperm count and motility (Fig. 2G-H). Consistent with these results, HE staining revealed a marked reduction in the sperm density within the epididymal cauda of the mice in the dAGEs group, and omega-3 supplementation reversed these changes. HE staining of the testes from the dAGEs group revealed atrophy of the seminiferous tubule epithelium, including thinning of the tubular walls, diminished cellularity, and visible shedding of germ cells into the tubular lumen. In contrast, the omega-3-supplemented group exhibited orderly seminiferous tubules and a resurgence of germ cells, and the diameters of the seminiferous tubules and the thickness of their epithelium were also restored (Fig. 2I). Quantitative assessment of seminiferous tubule diameter and epithelial thickness also indicated that dAGEs significantly impaired the thickness of the seminiferous tubules (Fig. 2J-K). Omega-3 supplementation could effectively mitigate the detrimental effects of dAGEs on testicular morphology. Furthermore, PAS staining revealed that exposure to dAGEs did not lead to the arrest of spermatogenesis, but caused epithelial thinning and fewer spermatozoa in the lumen, with some germ cell shedding (Fig. S1G). Omega-3 supplementation partially mitigated these defective effects. The results indicate a decrease in the number of c-Kit+ and SYCP3+ cells per seminiferous tubule in the dAGEs group, suggesting that AGEs may indeed cause phenotypic damage to spermatogenesis (Fig. S1H-J). Omega-3 treatment partially restores the number of c-Kit+ and SYCP3+ cells, which supports the protective role of omega-3 against AGE-induced damage. AGEs have been identified as critical mediators in chronic degenerative diseases associated with cellular senescence 44. We observed that dAGEs accelerated senescence in the spermatogenic microenvironment, as evidenced by the increased abundance of the well-established senescence markers P16 and P21 (Fig. 2L-N). This senescence-promoting effect was mitigated by supplementation with omega-3, which reduced the expression of P16 and P21. The senescence-associated secretory phenotype (SASP), which is secreted by senescent cells, promotes a chronic inflammatory microenvironment. We measured the levels of SASP-related factors in the testes of the mice. The results suggested an increase in the secretion of SASP-related factors in the testes of the mice in the dAGEs group. Then, following supplementation with omega-3, the secretion of SASP-related factors was significantly reduced (Fig. 2O). Previous studies have shown that AGEs are closely related to ROS 46-48. Figure 2P shows that ROS were overproduced in the testes of mice in the dAGEs group, but ROS production was significantly suppressed in the omega-3-treated group. The accumulation of ROS can activate intracellular signaling pathways that lead to apoptosis. Compared with that in the CTL group, the number of TUNEL-positive cells, which were predominantly localized to the basal region of the seminiferous tubules, was significantly greater in the dAGEs group. With omega-3 supplementation, the number of TUNEL-positive cells was substantially reduced (Fig. 2Q).

These findings indicate that excessive intake of AGEs disrupts spermatogenesis in mice, whereas omega-3 supplementation can facilitate the restoration of spermatogenesis. dAGEs accelerate cellular senescence within the seminiferous tubules of the testis, and this effect is accompanied by increased SASP secretion, oxidative stress, and apoptosis. Omega-3 supplementation mitigates these adverse effects.

### Omega-3 alleviates AGE-induced Sertoli cell injury and dysfunction *in vivo*

**Figure 3:**
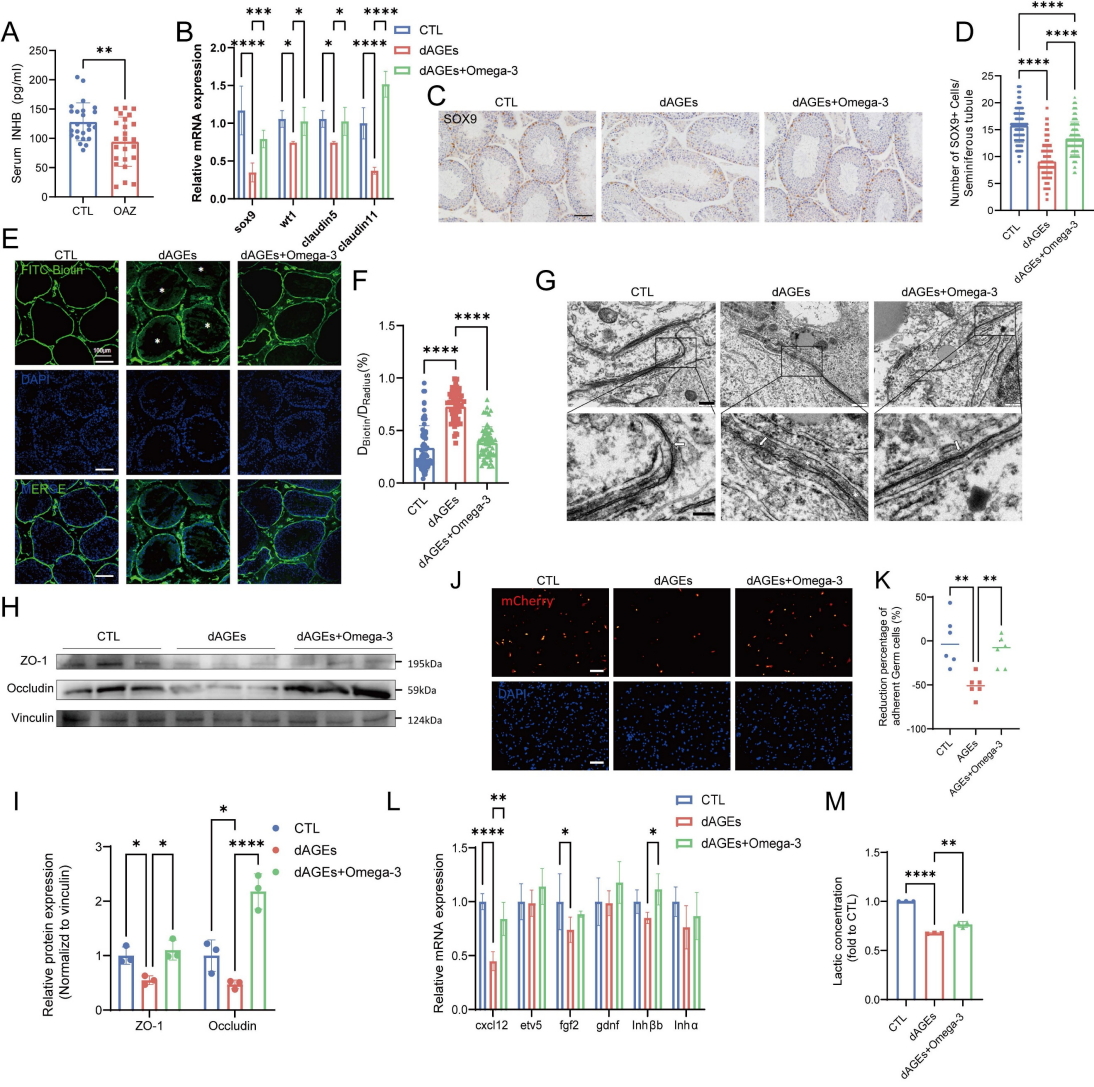
** Omega-3 alleviates AGE-induced Sertoli cell injury and dysfunction* in vivo*.** (A) Serum inhibin B levels were measured in CTL and OAZ patients. (B) The mRNA levels of Sertoli cell markers of mouse testis. (C) Immunohistochemistry staining of SOX9 protein expression. Scale bars: 50 μm. (D) Statistical analysis of the number of SOX+ cells in each seminiferous tubule across different groups. (E-F) Representative fluorescence images (E) of BTB integrity detected by the biotin tracer assay. The white asterisks (*) indicate the permeation of biotin into the seminiferous lumen. Scale bars: 100 μm. Bar graph (F) illustrating the extent of BTB damage, which was calculated randomly from 50-60 seminiferous tubules for each group. (G) The ultrastructure of tight junctions was observed using transmission electron microscopy. White arrows indicate the tight junctions of Sertoli cells and white asterisks denote discontinuous and loose structures. (H, I) The protein expression of ZO-1 and Occludin in mice testis was detected using Western blotting (H) and quantified by ImageJ (I). (J, K) The adhesion function of Sertoli cells was assessed using fluorescence detection(J) after co-culturing mCherry-labeled germ cells with Sertoli cells, and the resulting ratio of germ cells (GCs) to Sertoli cells (SCs) was quantified (K). Scale bars: 100 μm. (L)The mRNA levels of cytokines which influence germ cells in the testis of mice across different groups. (M) Lactate levels in the testis of mice across different groups and normalized to CTL. Statistical analysis between multiple groups was performed by one-way ANOVA. A two-sided p-value < 0.05 was considered to be statistically significant. The level of significance defined as p < 0.05 (*), p < 0.01 (**), p < 0.005 (***), p < 0.001 (****).

INHB, which is a crucial hormone that is produced by Sertoli cells, was present at significantly lower levels in the sera of OAZ patients (Fig. 3A). Similarly, INHB levels were significantly reduced in the sera of mice that were treated with dAGEs (Fig. S2A). Measurement of the mRNA levels of various cell markers in the seminiferous tubule epithelium further confirmed that Sertoli cells are the primary cell type affected by dAGEs in the spermatogenesis microenvironment (Fig. S2B). Our results demonstrated that dAGEs downregulated specific Sertoli cell markers, including sox9, wt1, claudin5, and claudin11, at the mRNA level. These effects were effectively mitigated by omega-3 supplementation (Fig. 3B).

The statistical analysis of the number of SOX9-positive cells in the seminiferous tubules of each group (Fig. 3C-D), as well as Western blotting analysis of SOX9 protein expression in testicular tissue (Fig. S2C-D), indicated that dAGEs cause significant damage to Sertoli cells. However, omega-3 rescued Sertoli cells. Moreover, analysis of single-cell data from the Human Protein Atlas (HPA) online database revealed that RAGE is highly expressed in Sertoli cells (Fig. S2E). Therefore, Sertoli cell dysfunction may play a role in the process of dAGE-induced OAZ.

We investigated the function of Sertoli cells in the BTB and spermatogenesis. In mice in the dAGEs group, BTB function was impaired, leading to the leakage of biotin into the seminiferous lumens (Fig. 3E-F). The TEM analysis of the testis revealed that in the dAGEs group, the intercellular junctions between the Sertoli cells were discontinuous, disrupted, and structurally disordered (as indicated by the arrows), indicative of a compromised BTB (Fig. 3G), and these tissues had downregulated tight junction proteins (Fig. 3H-I). Sertoli cells maintain adhesion junctions with different stages of germ cells, effectively anchoring germ cells within the germinal niches 49, 50. In co-culture experiments, dAGEs decreased the adhesion of germ cells to Sertoli cells (Fig. 3J-K, Fig. S2F). Additionally, dAGEs reduced the mRNA levels of key Sertoli cell secretory factors (CXCL12 and FGF2) and the secretion of lactate, impairing spermatogonial renewal and differentiation 51-53 (Fig. 3L-M). Omega-3 supplementation restored the integrity of the BTB and upregulated tight junction proteins. Omega-3 supplementation partially restored the levels of secretory factors (such as CXCL12) and lactate. Moreover, in the omega-3-treated group, more germ cells were observed to adhere to Sertoli cells.

These findings highlight the critical role of Sertoli cell dysfunction in dAGE-induced spermatogenic impairment and reveal that omega-3 alleviates AGE-induced injury to and dysfunction of Sertoli cells *in vivo*.

### Omega-3 alleviates AGE-induced senescence and dysfunction in TM4 cells *in vitro*

**Figure 4:**
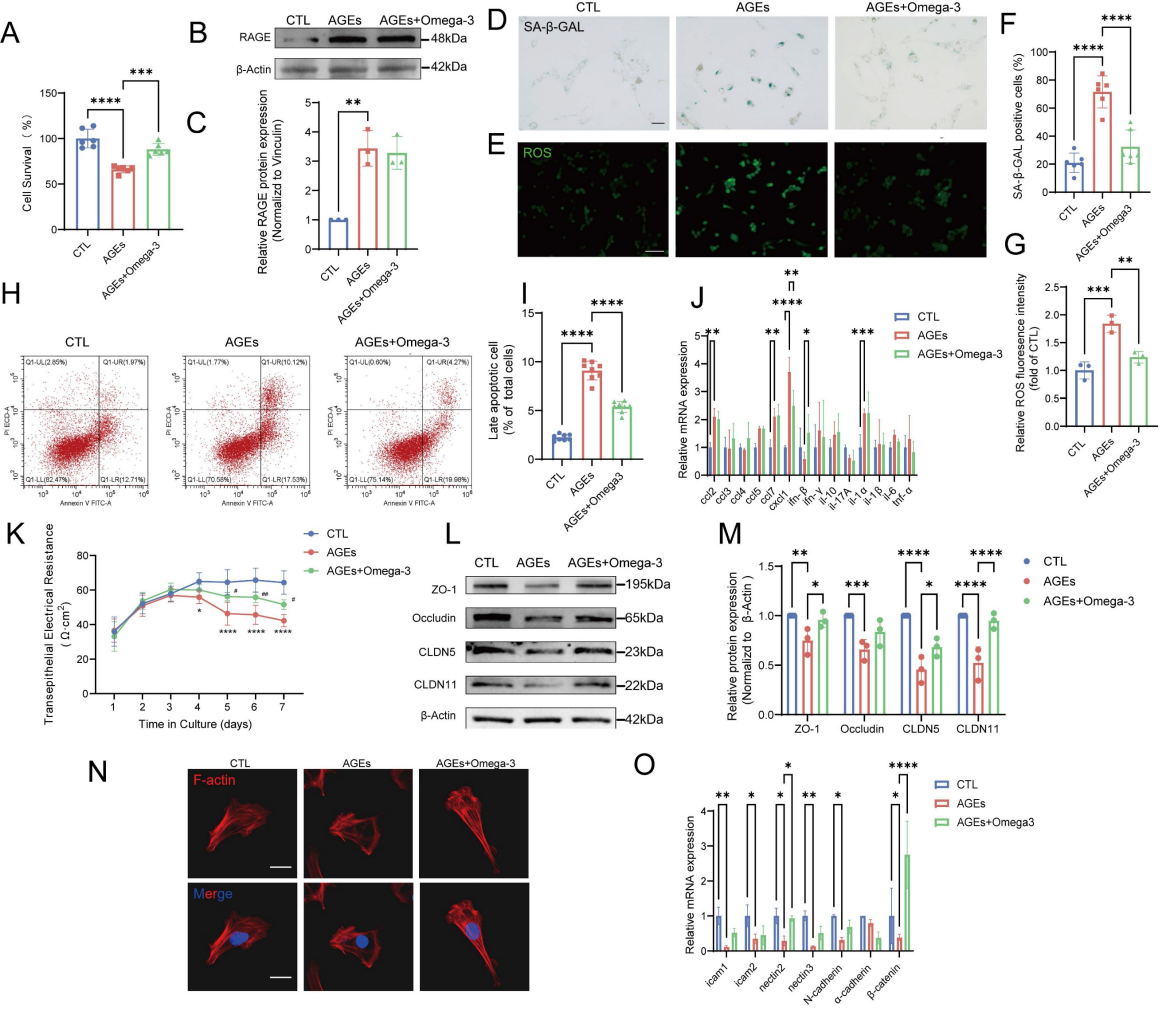
** Omega-3 alleviates AGE-induced senescence and dysfunction in TM4 cells* in vitro.*** (A) Analysis of TM4 cell survival following treatment with AGEs at a concentration of 200 μg/mL, and the rescuing effects of Omega-3, as determined by the CCK-8 assay. (B-C) The protein expression of RAGE in TM4 cells was detected using Western blotting following treatment with AGEs and an Omega-3 supplement, and quantified by ImageJ (C). (D, F) SA-β-gal activity detection usingβ-galactosidase staining, and statistical analysis of the proportion of positive cells. Scale bar: 50 μm. (E, G) ROS detection using DCFH-DA staining and the fluorescence densities were calculated using ImageJ. Scale bar: 50 μm. (H, I) Analysis of apoptosis using Annexin V-FITC staining by flow cytometry. The late apoptotic rates were calculated and presented in the right panel. (J) The mRNA levels of SASP factors in TM4 cells. (K)Assessment of TM4 cell barrier integrity* in vitro*. After cell barrier formation, cells were treated and TER detection were used to analyze the integrity of TM4 cell barriers. (L, M) Tight junction protein levels in TM4 cells were examined by western blotting, and quantified by ImageJ (M). (N) Representative images of the formation of cytoskeleton in TM4 cells by F-actin staining immunofluorescence. Scale bar: 20 μm. (O) The mRNA levels of cell adhesion function-related genes in TM4 cells. Statistical analysis between multiple groups was performed by one-way ANOVA. A two-sided p-value < 0.05 was considered to be statistically significant. The level of significance defined as p < 0.05 (*), p < 0.01 (**), p < 0.005(***), p < 0.001 (****).

*In vitro* experiments using the TM4 cell line revealed that cell viability was significantly decreased with increasing AGEs-BSA concentration (0-200 μg/mL) and stabilized at approximately 50% following treatment with 200-800 μg/mL AGEs-BSA (Fig. S3A). The protein expression of RAGE also significantly increased after treatment with 200 μg/mL AGEs-BSA (Fig. S3B). Omega-3 (200 µM) enhanced the viability of AGEs-treated cells but had a minimal effect on RAGE protein levels (Fig. 4A-C).

SA-β-gal activity is a key indicator of cellular senescence, and SA-β-gal activity changes in patterns that are similar to changes in ROS levels; that is, SA-β-gal activity increases after AGE-induced damage, and this change can be reversed by omega-3 (Fig. 4D-G). Moreover, in the AGE group, the MDA levels were increased, and the SOD activity was significantly impaired, while omega-3 supplementation reduced the MDA levels and restored SOD activity (Fig. S3C-D). The flow cytometry analysis results indicated that omega-3 could alleviate the TM4 cell apoptosis induced by AGEs (Fig. 4H-I). The mRNA levels of multiple proinflammatory cytokines and chemokines associated with the SASP (IL-1α, CCL2, CCL7, and CXCL1) were increased in TM4 cells with AGE-induced senescence, possibly by affecting neighboring cells and the testicular microenvironment (Fig. 4J). These results indicate that AGEs exposure can trigger senescence in TM4 cells by impairing antioxidant mechanisms, causing apoptosis and exacerbating oxidative stress. Additionally, omega-3 potentially exerts protective effects against AGE-induced senescence in TM4 cells. Omega-3 treatment mitigated these effects by reducing SA-β-gal activity, ROS levels, and MDA levels while restoring SOD activity and alleviating apoptosis in TM4 cells, demonstrating its potential protective effects against AGE-induced senescence.

We studied the function of TM4 cells *in vitro* to focus on the effects of AGEs, thus avoiding *in vivo* complexities. TER assays revealed that, compared with the control, AGEs significantly decreased electrical resistance, and omega-3 treatment protected cell barriers (Fig. 4K). Additionally, AGE-induced reductions in BTB tight junction proteins (ZO-1, Occludin, CLDN5, and CLDN11) were mitigated by omega-3 treatment (Fig. 4L-M). The main component of the cytoskeleton, namely, F-actin, provides attachment sites for BTB-related proteins, and it is essential for adhesion between Sertoli cells and germ cells 54, 55. AGEs caused F-actin aggregation in TM4 cells, whereas omega-3 restored F-actin arrangement (Fig. 4N). AGEs also decreased the mRNA levels of adhesion-related molecules, whereas omega-3 supplementation restored the mRNA levels of nectin2 and β-catenin (Fig. 4O). These results indicate that omega-3 can improve Sertoli cell dysfunction caused by AGEs, which is consistent with the *in vivo* findings.

### Omega-3 suppresses the AGE-induced increase in MERCs in Sertoli cells

**Figure 5:**
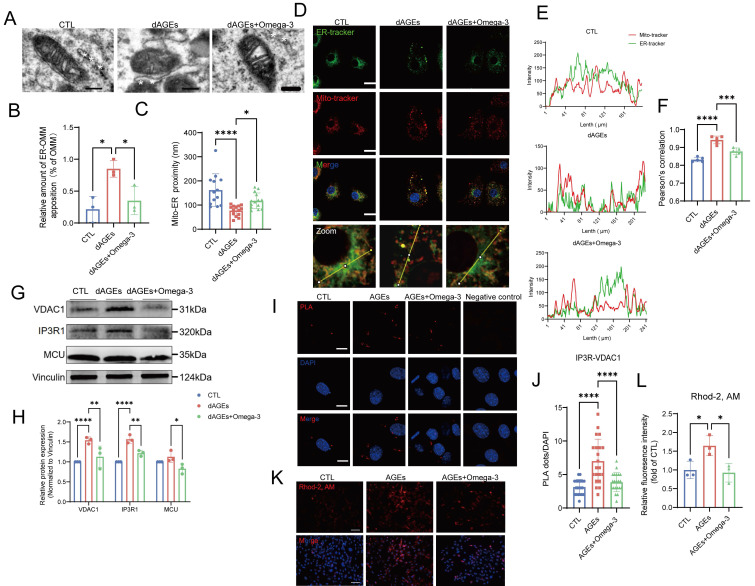
** Omega-3 suppresses the AGE-induced increase in MERCs in Sertoli cells.** (A-C) Representative transmission electron microscopy (TEM) images of Mito and ER in Sertoli cells of mice testes from different groups (A). The white asterisks (*) indicate the ER. Quantification of the extent of MERCs (<100 nm) (B) and the Mito-ER proximity in Sertoli cells of mice testis from different groups (n= 12 to 15 mitochondria in 5 fields per condition) (C). (D-F) Representative immunostaining pictures of colocalization between mitochondria and ER in primary Sertoli cells from different groups mice (D). Mitochondria and ER are marked with Mito-tracker and ER-tracker. Scale bar, 10 μm (E) Line intensity profile analysis of Mito-tracker and ER-tracker. Intensity values were measured along a path indicated in lower pictures. The Pearson's correlation between ER-tracker and Mito-tracker was analyzed (F). (G, H) MERCs protein levels in treated TM4 cells were examined by western blotting, and quantified by ImageJ. (I, J) Representative images of proximity ligation assay (PLA) targeting IP3R3-VDAC1 interactions (I) and quantification of the PLA red fluorescent dots (J) in treated TM4 cells (n= 20 to 22 cells in 10 fields per condition). Scale bar: 10 μm. (K, L) Mitochondrial Ca^2+^ detection using Rhod-2 staining (K) and the fluorescence densities were calculated using ImageJ and normalized (L). Statistical analysis between multiple groups was performed by one-way ANOVA. A two-sided p-value < 0.05 was considered to be statistically significant. The level of significance defined as p < 0.05 (*), p < 0.001 (****).

TEM examination of Sertoli cells in dAGEs-treated mouse testes revealed mitochondrial shrinkage, crista loss, vacuolization, and diminished ribosome numbers and absent lamellae in the ER (Fig. 5A). Considering that these simultaneous structural changes in the mitochondria and ER, as well as MERCs, are involved in the regulation of cellular oxidative stress and senescence 56, 57, we hypothesized that MERCs are an important link in Sertoli cells with AGE-induced damage. We observed that Sertoli cells in dAGEs-treated mice exhibited decreased mitochondria-ER distances, with a significantly greater proportion of mitochondria establishing close contacts with the ER (Fig. 5B-F).

This greater proportion of contact was also observed in AGEs-treated TM4 cells (Fig. S4A-C). AGEs stimulation of TM4 cells led to notable increases in the transcription and protein levels of key proteins of MERCs (Fig. S4D, Fig. 5G-H), IP3R1 and VDAC1. These proteins form a complex that primarily regulates the transfer of calcium ions from the ER to the mitochondria 58. In situ PLA revealed a notable increase in the level of the IP3R1-VDAC1 complex in AGEs-treated TM4 cells (Fig. 5I-J). Additionally, an increase in mitochondrial Ca^2+^ load was observed following AGEs exposure (Fig. 5K-L). Furthermore, AGEs significantly enhance mitochondrial Ca^2+^ signaling amplitude in TM4 cells following ATP stimulation (Fig. S4E). This effect originates from the overactivation of IP3R channels, which can be effectively reversed by the IP3R inhibitor 2-APB. Omega-3 intervention partially suppresses the pathological activation of calcium channels without interfering with ATP-mediated acute Ca^2+^ signaling responses, indicating its selective modulation of basal calcium homeostasis while preserving physiological signal transduction. The mitochondrial permeability transition pore (mPTP), situated within the inner mitochondrial membrane (IMM), is a voltage-dependent, nonselective channel. A previous study indicated that excessive calcium accumulation within mitochondria can trigger mPTP opening 59. Following treatment with AGEs, we observed a pathological increase in mPTP opening in TM4 cells (Fig. S4 F-G). We infer that the release of Ca^2+^ through the mPTP is a potential cause of increased intracellular calcium levels under conditions of AGEs-driven TM4 cell damage. Furthermore, there was a noted elevation in intracellular calcium levels in cells treated with AGEs, which was reflected by ratio-type dual-wavelength dye Fura-10 (Fig. S4 J-K). This result suggests that the release of Ca^2+^ through the mPTP is also a potential cause of increased intracellular calcium levels.

Interestingly, the protective effect of omega-3 against AGE-induced damage is also mediated by alleviating the excessive activation of MERCs. Omega-3 restored the distance between mitochondria and the ER, decreased MERC sites, and reduced the levels of IP3R1 and VDAC1 and their complexes, thereby reestablishing the homeostasis of mitochondria and intracellular Ca^2+^. Thus, we conclude that an AGEs-rich diet induces Sertoli cells to establish more MERCs both *in vitro* and *in vivo*, which is reduced by omega-3.

### Omega-3 ameliorates AGE-induced ER stress and mitochondrial dysfunction

**Figure 6:**
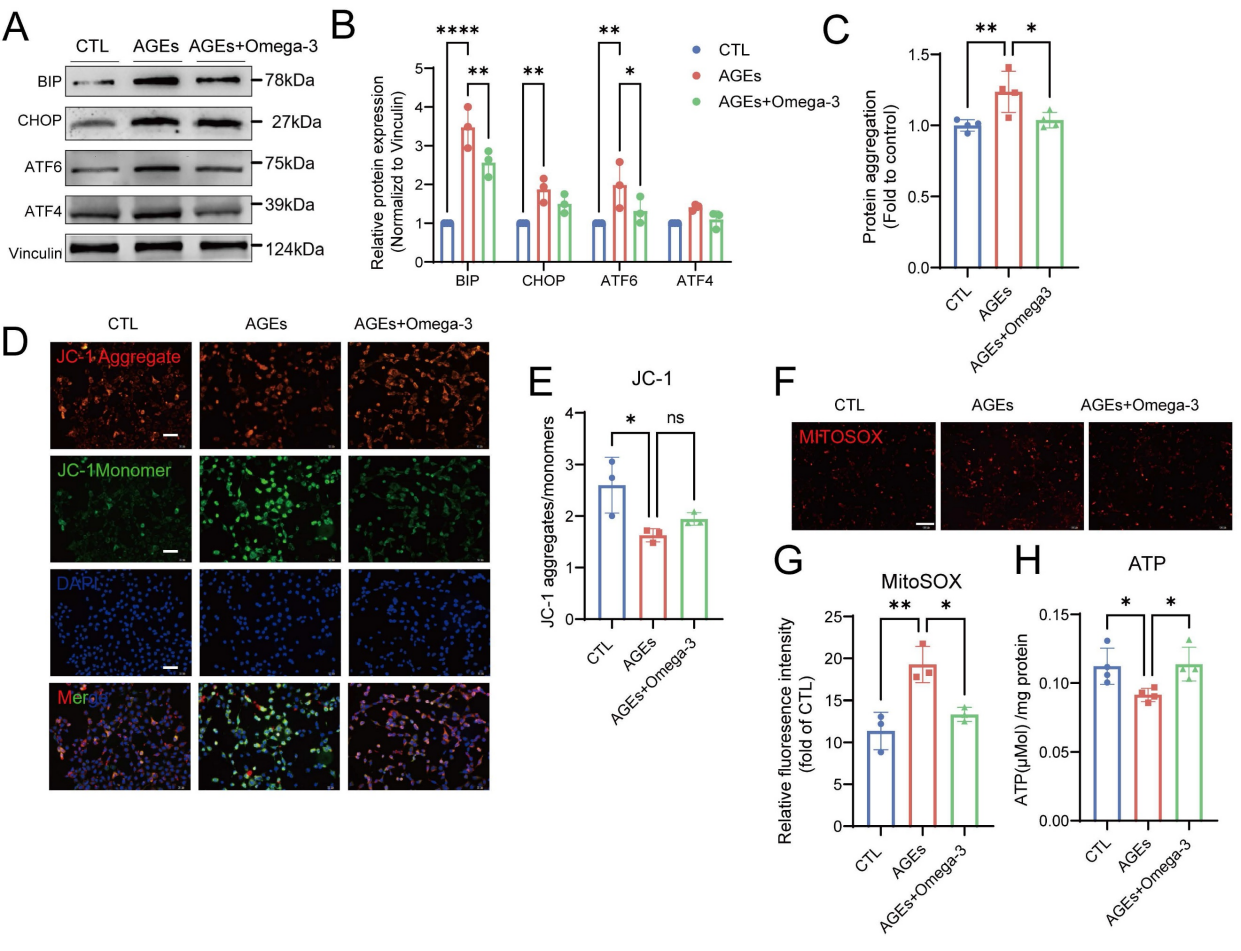
** Omega-3 ameliorates AGE-induced ER stress and mitochondrial dysfunction.** (A, B) ER stress protein levels in treated TM4 cells were examined by western blotting, and quantified by ImageJ. (C) Protein aggregation was evaluated using PROTEOSTAT^®^ Protein Aggregation Kit and presented as relative fluorescence units and normalized to CTL level. (D, E) Mitochondrial membrane potential (Δψm) in treated TM4 cells was detected using JC-1 staining (D). Representative images of JC-1 aggregates (red) and JC-1 monomers (green) The ratio of JC-1 aggregates to JC-1 monomers was calculated using ImageJ (E). (F, G) Representative images of MitoSOX (red) fluorescence in treated TM4 cell (F). Scale bar: 100 μm Relative MitoSOX fluorescence was quantitatively analyzed (G). (H) The ATP activity in TM4 cells weas normalized per mg of protein. Statistical analysis between multiple groups was performed by one-way ANOVA. A two-sided p-value < 0.05 was considered to be statistically significant. The level of significance defined as p < 0.05 (*), p < 0.01 (**).

Disruptions of MERCs destabilize intracellular homeostasis, impairing ER and mitochondrial function 60. In the ER, the levels of proteins that are involved in the unfolded protein response significantly increased after the addition of AGEs, and these protein levels were restored following supplementation with omega-3 (Fig. 6A-B). The accumulation of unfolded or misfolded proteins that cannot be properly folded into their secondary structures leads to the formation of protein aggregates within the cell 61. AGEs also increased the number of intracellular protein aggregates in TM4 cells, as detected using a small-molecule fluorescent probe, and this effect was suppressed by omega-3 (Fig. 6C).

In response to AGEs, mitochondrial function in TM4 cells was impaired, as characterized by decreased mitochondrial transmembrane potential (Fig. 6D-E), elevated MitoSOX fluorescence (Fig. 6F-G), and reduced ATP synthesis (Fig. 6H). These results indicate increased oxidative stress and dysfunction in the mitochondria. Omega-3 restored normal mitochondrial function. This evidence confirmed the protective effects of omega-3 in suppressing AGE-induced ER stress and mitochondrial dysfunction.

### RAGE is a central mediator of AGE-induced Sertoli cell dysfunction

**Figure 7:**
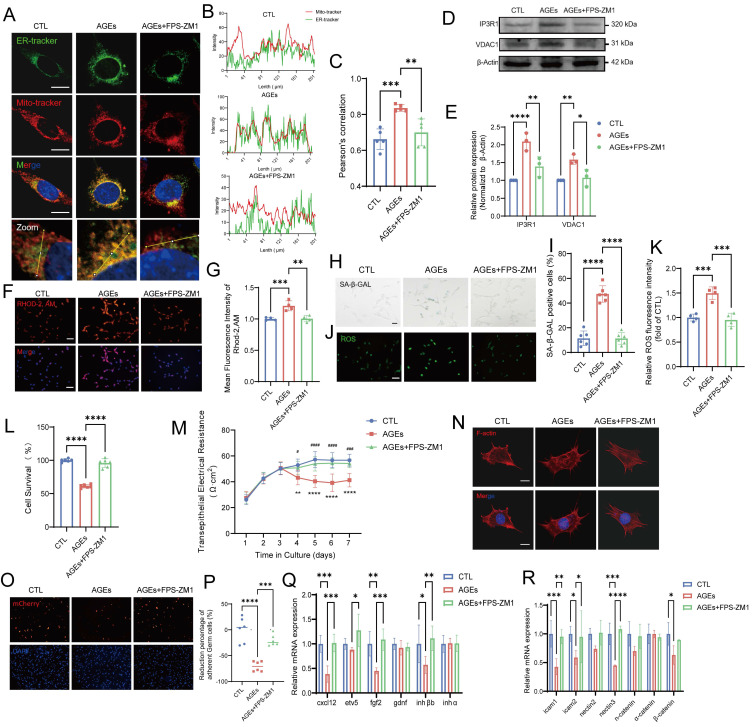
** RAGE is a central mediator of AGE-induced Sertoli cell dysfunction.** (A-C) Representative immunostaining pictures of colocalization between mitochondria and ER in AGEs treated TM4 with or without the RAGE inhibitor FPS-ZM1(A). Scale bar: 10 μm. Line intensity profile analysis of Mito-tracker and ER-tracker (B). Intensity values were measured along a path indicated in lowest pictures (A). The Pearson's correlation between ER-tracker and Mito-tracker was analyzed (C). (D, E) MERCs protein levels in treated TM4 cells were examined by western blotting. (F, G) Representative immunostaining pictures of mitochondrial Ca^2+^ detection in treated TM4 (F) and the fluorescence densities were calculated using ImageJ and normalized (G). (H, I) SA-β-gal activity detection usingβ-galactosidase staining, and statistical analysis of the proportion of positive cells. Scale bar: 50 μm. (J, K) ROS detection using DCFH-DA staining and the fluorescence densities were calculated using ImageJ. Scale bar: 50 μm. (L) Analysis of TM4 cell survival following treatment with AGEs with or without FPS-ZM1, as determined by the CCK-8 assay. (M) Assessment of TM4 cell barrier integrity* in vitro*. After cell barrier formation, cells were treated and TER detection were used to analyze the integrity of TM4 cell barriers. (N) Representative images of the formation of cytoskeleton in TM4 cells by F-actin staining immunofluorescence. Scale bar = 20 μm. (O, P) The adhesion function of Sertoli cells was assessed using fluorescence detection (O) after co-culturing mCherry-labeled germ cells with Sertoli cells, and the resulting ratio of germ cells (GCs) to Sertoli cells (SCs) was quantified (P). (Q) The mRNA levels of cytokines which influence germ cells in TM4 cells. (R) The mRNA levels of cell adhesion function-related genes in TM4 cells. Statistical analysis between multiple groups was performed by one-way ANOVA. A two-sided p-value < 0.05 was considered to be statistically significant. The level of significance defined as p < 0.05 (*), p < 0.01 (**), p < 0.005(***), p < 0.001 (****).

The intracellular effects of AGEs are predominantly mediated by cell surface receptors such as RAGE, which is a key component of the AGE-RAGE axis 62. After the addition of the RAGE inhibitor FPS-ZM1 (10 μM), the AGE-induced increase in RAGE protein expression was suppressed, and RAGE expression was restored to normal levels (Fig. S5A). The AGE-induced increase in the colocalization of mitochondria and the ER, as well as the elevated levels of the key MERC proteins IP3R1 and VDAC1, were restored to normal after treatment with FPS-ZM1 (Fig. 7A-E). Additionally, the mitochondrial calcium overload caused by the AGE-induced increase in MERCs was completely reduced to normal levels after treatment with FPS-ZM1 (Fig. 7F-G). The downstream characteristics of MC disruption, including impaired ER and mitochondrial function, were suppressed by the blocking action of FPS-ZM1 (Fig. S5B-D).

Moreover, the increased SA-β-gal staining and elevated ROS levels, which are markers of AGE-induced senescence in TM4 cells, as previously confirmed, were reversed after blocking the action of receptors such as RAGE (Fig. 7H-K). The results of the CCK-8 assay revealed that the AGE-induced decrease in TM4 cell viability was significantly increased by FPS-ZM1 (Fig. 7L). Additionally, FPS-ZM1 partially restored the barrier, secretory, and adhesive functions of Sertoli cells damaged by AGEs (Fig. 7M-R, Fig. S5E). These findings confirm that the increase in MERC numbers, cellular senescence, and functional impairment in Sertoli cells are directly mediated by AGEs via the RAGE pathway, thereby elucidating the mechanism underlying the effects of AGEs.

Further investigation was conducted to compare protective effects of omega-3, FPS-ZM1, and their combination on AGEs-damaged TM4 cells, as assessed by cell viability and ROS levels (Fig. S5F-H). Omega-3 showed a modestly greater protective effect than FPS-ZM1, whereas the combination therapy failed to show a synergistic protection compared to using omega-3 monotherapy. This may imply that omega-3 has already comprehensively encompassed the pathways targeted by FPS-ZM1, and these results suggest that omega-3 exerts a protective effect against the damage signals activated by AGEs via RAGE. However, other pathways affected by omega-3, such as antioxidant and anti-inflammatory pathways, may also exert indispensable effects.

## Discussion

The impact and mechanisms of AGEs on reproductive health, particularly fertility, require further investigation. Our study revealed elevated serum AGEs levels in patients with OAZ, and diets rich in AGEs can cause sperm generation disorders in mice. Mechanistically, AGEs enter Sertoli cells through RAGE, leading to increased MERC numbers and disruption of calcium ion homeostasis. These changes subsequently trigger ER stress and mitochondrial damage in Sertoli cells, ultimately leading to Sertoli cell senescence and apoptosis. Subsequently, injured Sertoli cells exhibit oxidative stress imbalance and increased SASP secretion within the spermatogenic microenvironment. Moreover, these alterations impair the barrier, adhesion, and secretory functions of Sertoli cells, culminating in spermatogenesis disorders. Dietary omega-3 supplementation can mitigate AGE-induced spermatogenic dysfunction, mainly by regulating MERCs to restore function, without reducing local AGEs content or downregulating RAGE levels. RAGE, which is a critical receptor of AGEs, has been proven to play a role in mediating the detrimental effects of AGEs, including increasing the number of mitochondria-ER contacts and causing Sertoli cell senescence.

OAZ has multifactorial causes. In modern society, unhealthy lifestyles and metabolic disorders are increasingly becoming significant inducers of male spermatogenic disorders 63. Previous studies have shown that elevated AGEs levels play a crucial role in the impact of certain metabolism-related diseases on male spermatogenic function, such as the decreases in sperm parameters that are caused by diabetes and obesity 16, 64, 65. However, these diseases often accompany by increased AGEs levels. Since AGEs are not a routine clinical indicator for spermatogenic disorders, they are not usually a focus in patients with OAZ. Currently, there is insufficient evidence to directly link elevated AGEs levels with OAZ, and the role of AGEs in OAZ requires more scientific research to explore and confirm. On the basis of our clinical data, serum AGEs levels are increased in patients with OAZ. However, regarding limitations due to small sample sizes or potential selection bias, future studies may consider including a larger number of clinical samples to provide stronger support for our inference. Additionally, multicenter studies may help enhance the generalizability and reliability of the results.

Under normal physiological conditions, the accumulation of endogenous AGEs occurs at a very slow rate. However, exogenous AGEs from the diet are the primary contributors to AGEs accumulation in blood and tissues, often leading to pathological conditions 66. Exogenous AGEs primarily originate from high-temperature cooking methods, such as baking, frying, and stir-frying, and AGEs intake has significantly increased with the consumption of modern diets. In our study, we used AIN-93G feed that had been heated at high temperatures for a prolonged period of time to investigate the mechanisms and therapeutic possibilities of dAGE-induced dysfunction of sperm generation in mice. The literature has confirmed that prolonged heating can significantly increase the AGE content in mouse feed 67. We detected increased AGEs levels in the sera and testes of dAGEs-fed mice, along with a marked decrease in sperm concentration and motility. The sperm parameters are consistent with the results of previously published studies 68-70. We conducted histological analysis of testes from mice in the dAGEs group, and we observed atrophy of the seminiferous tubule epithelium and detachment of germ cells from the basement membrane through HE staining. All these phenotypes are similar to previous OAZ findings 71. Additionally, we detected an oxidative stress imbalance in the testicular seminiferous tubules, as well as a significant increase in the levels of the senescence markers P16 and P21. These results suggest that AGEs may cause significant abnormalities in the testicular spermatogenic microenvironment, thereby affecting sperm generation and function.

Furthermore, we detected a significant decrease in INHB levels in both the sera of OAZ patients and mice in the dAGEs group. Since Sertoli cells are the sole source of INHB, the results suggested that we should focus on changes in Sertoli cell function. We detected a decrease in the number of Sertoli cells and damage to their barrier, adhesion, and secretory functions. These findings emphasize the key role of Sertoli cells in AGE-induced spermatogenic disorders. Recent studies have shown that AGEs significantly inhibit testosterone production in rat Leydig cells following treatment with human chorionic gonadotropin 72. Similarly, in our study, mice in the dAGEs group exhibited decreased serum testosterone levels. Additionally, adverse effects of AGEs on mouse sperm have been reported, including reduced sperm motility and increased DNA damage 68. Furthermore, RAGE is expressed in Sertoli cells, spermatogonia, and Leydig cells. The damage caused by AGEs to cells within the seminiferous tubules appears to be widespread. However, these findings do not contradict our findings that emphasize the key role of Sertoli cells in AGE-induced spermatogenic disorders. Supplementation with omega-3 significantly restored sperm concentration, motility, and INHB levels in mice, indicating a reversal of Sertoli cell damage and a positive effect on spermatogenic function. Although omega-3 did not significantly alter AGEs levels in sera and testes (Fig. 2B-C), it improved sperm quality and mitigated testicular senescence induced by dAGEs. This suggests that omega-3's beneficial effects are mediated through modulating the downstream impacts of AGEs, rather than by reducting in AGEs levels.

The appearance of aging features and functional disorders in Sertoli cells shows that AGEs play a key role in promoting cellular aging. Cellular senescence often manifests as proliferation arrest under stress conditions, which is accompanied by the secretion of SASP-related factors 73. Additionally, the specific increase in ROS levels has been proven to play a potentially key role in inducing and maintaining the process of cellular senescence 74. On the basis of these findings, we further explored and revealed a series of downstream effects triggered by Sertoli cell senescence, including mainly enhanced SASP, increased apoptosis, and oxidative stress imbalance.

Aging cells can continuously produce characteristic pathogenic SASP factors, which not only disrupt tissue homeostasis but also lead to pathological changes in tissue function and loss of regenerative capacity, thereby driving the spread of secondary aging and creating a proinflammatory environment 75. The mRNA levels of SASP-related factors were increased in AGEs-treated TM4 cells, which was consistent with the increase in SASP-related factors, including IL-6, in the testes of AGE-induced mice. Similarly, some studies suggest that AGEs can indeed increase cellular SASP-related factor secretion 76. On the basis of these results, we believe that the AGE-induced senescence of Sertoli cells is characterized by increased SASP-related factor secretion, causing widespread cellular senescence in the seminiferous tubules and significantly impacting the testicular microenvironment and sperm generation function. For a long time, cellular senescence and apoptosis have been considered to be two completely different cell fates, with senescent cells typically showing resistance to apoptosis. However, recent research has shown that apoptosis and senescence may be regulated by similar mechanisms involving mitochondria-dependent pathways 77. During the testicular aging process, the apoptosis of Sertoli cells leads to the destruction of seminiferous tubule structure and a decrease in spermatogenic function, thereby affecting spermatogenic function 78, 79. In our study, flow cytometry revealed an increased proportion of late apoptotic AGEs-treated TM4 cells, as well as an increase in the number of TUNEL-positive cells in the seminiferous tubules, which confirmed the link between AGE-induced cellular senescence and apoptosis.

AGEs are key factors in promoting ROS generation 8, and ROS are also important inducers of the SASP and apoptosis 80. Oxidative stress imbalance plays a significant role in male infertility 81. In our study, we assessed the efficacy of the antioxidant system by measuring ROS levels, antioxidant enzyme (SOD) activity, and the level of the oxidative stress biomarker MDA. These results support the idea that oxidative stress plays a significant negative role in AGE-induced dysfunction of sperm generation. The most likely sources of this ROS production are the mitochondria and the ER 82, 83. In our study, we observed that AGEs affected TM4 cells, leading to impaired mitochondrial ATP synthesis and changes in the mitochondrial membrane potential. The significant increase in MitoSox further confirmed the increase in ROS production in mitochondria, which disrupted cellular oxidative stress homeostasis. In our study, the expression of ER stress-related proteins in TM4 cells were significantly elevated after AGEs treatment, accompanied by the activation of the unfolded protein response and the exacerbation of protein aggregation. These findings indicate that mitochondrial dysfunction and ER stress together lead to abnormal increases in ROS.

MERCs are sites of physical connection between the ER and mitochondria, and they are involved in regulating ROS production. MERCs are not fixed but change according to pathological conditions, and increasing or decreasing their contact area directly affects the metabolic state of organelles 84. In our study, we found for the first time that MERC numbers in Sertoli cells were abnormally increased after AGEs treatment, and the expression of MAM-related proteins increased. These findings suggest that AGEs may exert a direct impact on MERC dynamics, potentially disrupting the balance and communication between the ER and mitochondria. MERCs are crucial for maintaining calcium homeostasis because they facilitate the movement of calcium from the ER to the mitochondria 85. The maintenance of mitochondrial calcium homeostasis largely depends on MAMs, with estimates suggesting that over 80% of mitochondrial Ca²⁺ is acquired from ER 86. We further revealed that the abnormal increase in the number of MERCs resulted in mitochondrial Ca^2+^ overload. The subsequent opening of the mPTP may be a potential cause of elevated intracellular calcium levels overall. This suggests that AGEs affect the calcium conduction function of MERCs. We further revealed that mitochondrial dysfunction and increased ER stress occurred after AGEs treatment. Under pathological conditions of excessive AGEs levels, mitochondrial Ca²⁺ overload triggers mitochondrial collapse, and simultaneously, a cellular calcium imbalance leads to weakened protein folding capacity, inducing ER stress 87. The dynamic regulation of MERCs reflects their complex roles in cellular function. Recent studies underscore the pivotal role of MERCs in male fertility. A proteomic analysis revealed a high degree of conservation of MAM proteins in human and mouse testes, indicating their significance in spermatogenesis 88. Additionally, Guo et al. found that fluoride promotes calcium ion transfer through MAMs in spermatocytes, thereby affecting their function 89. In summary, MERCs are likely critical for preserving Sertoli cell function and supporting the spermatogenesis process, particularly under conditions of metabolic stress. These findings highlight the critical role of MERCs in maintaining cellular health and function and suggest their potential as intervention targets in the aging process. Also, in naturally aging mice, ER stress and mitochondrial dysfunction occurred in Leydig cells, but MERC numbers were reduced, which is inconsistent with our results, where MERC number increased after AGE treatment 60. Since natural aging is a multifactorial and multistage process involving various stressors, while high AGE level is a single stressor, this difference in MERC numbers may be due to different cellular stressors, and the different cell types maybe another possible reason. Moreover, as MERC function is a double-edged sword 90, this difference indicates that both excessive activation and deterioration can lead to cell senescence or death.

The regulation of the Sertoli barrier, adhesion, and secretory functions by MERCs can be explained, on the one hand, through their impact on Sertoli cell apoptosis; on the other hand, MERCs are closely related to the cytoskeleton, and changes in these structures may cause cytoskeletal disorders, affecting the BTB and adhesion. Moreover, disordered calcium ion signaling interferes with the secretory activities of Sertoli cells, leading to damage to secretory function 29. Similar to our study, an increasing number of articles have described the importance of the MAM proteome in cell biology and aging regulation 91-94. We believe that MAM proteins and their functions play crucial roles in AGE-induced senescence.

Owing to relatively fixed personal dietary habits, coupled with the broad and hidden pathways of AGEs intake, we cannot completely prevent their impact on the body by simply avoiding AGEs intake. Therefore, the identification of effective treatment approaches is particularly urgent. Omega-3, as health supplements, can be incorporated into of a daily diet. Omega-3 has powerful antioxidant and anti-inflammatory effects 95, and they have potential for use in treating AGE-induced spermatogenic disorders. In terms of mechanism, omega-3 can restore MERCs to normal levels after damage by AGEs. Omega-3 can regulate the function of calcium pumps (such as SERCA pumps) and calcium exchangers (such as Na⁺/Ca²⁺ exchangers) 96. By increasing the activity of calcium pumps, omega-3 helps pump calcium ions from the cytoplasm back into the ER for storage, thereby reducing the concentration of calcium ions in the cytoplasm and maintaining calcium ion homeostasis. When omega-3 are ingested, they are integrated into the lipid bilayers of cell membranes, increasing the space between membrane lipid molecules and improving membrane fluidity. The presence of multiple double bonds in omega-3 fatty acids causes the fatty acid chains to adopt a kinked conformation. Upon incorporation into the phospholipid bilayers, this particular molecular architecture endows lipid molecules with enhanced mobility, thereby boosting membrane fluidity 97. Recent studies have highlighted the impact of omega-3 fatty acids on erythrocyte membrane fluidity, and it is likely that subcellular organelle membranes are similarly regulated 98. This enhanced fluidity may contribute to the restoration of MERCs function. Our findings suggest a significant association between omega-3 fatty acids and the regulation of MERCs. Further investigation is required to determine the precise effects of omega-3 fatty acids on MERCs structure and their influence on intracellular signaling and metabolic processes. These findings suggest that omega-3 may regulate MERCs, although this remains to be confirmed. Thus, omega-3 could have potential for treating diseases that involve increased MERC numbers, with specific mechanisms warranting further investigation.

Our *in vivo* experiments demonstrated the therapeutic effect of omega-3 on AGE-induced spermatogenesis dysfunction in mice. However, due to limitations of exogenous AGEs impact, species differences, and fixed-dose diets, future research should consider execution of clinical trials of omega-3 in OAZ patients with high AGEs. Furthermore, delving into a more nuanced investigation of the downstream signaling pathways modulated by the remodeling of MERCs is also a highly promising research direction.

## Conclusion

Our study demonstrates that omega-3 fatty acids mitigate dietary AGE-induced Sertoli cell senescence and oligoasthenozoospermia through remodeling mitochondria-ER contacts, highlighting the AGE-RAGE axis as a potential therapeutic target for male fertility.

## Supplementary Material

Supplementary methods, figures and tables.

## Abbreviations

AGEs: Advanced Glycation End Products
AUC: Area Under the Curve
ER: Endoplasmic Reticulum
INHB: Inhibin B
IP3R1: Inositol 1,4,5-trisphosphate Receptor 1
MAM: Mitochondria-Associated Membranes
MERCs: Mitochondria-Endoplasmic reticulum Contacts
mPTP: Mitochondrial Permeability Transition Pore
OAZ: Oligoasthenozoospermia
PUFAs: Polyunsaturated Fatty Acids
RAGE: Receptor for AGE
ROS: Reactive Oxygen Species
SASP: Senescence-Associated Secretory Phenotype
TER: Transepithelial Resistance
dAGEs: Advanced Glycation End Product-Rich Diet
VDAC1: Voltage-dependent Anion Channel 1
Omega-3: Omega-3 Polyunsaturated Fatty Acids
BTB: Blood-testis Barrier

## References

[1] Agarwal A, Baskaran S, Parekh N (2021). Male infertility. Lancet.

[2] Huang B, Wang Z, Kong Y (2023). Global, regional and national burden of male infertility in 204 countries and territories between 1990 and 2019: an analysis of global burden of disease study. BMC Public Health.

[3] Łakoma K, Kukharuk O, Śliż D (2023). The Influence of Metabolic Factors and Diet on Fertility. Nutrients.

[4] Benatta M, Kettache R, Buchholz N (2020). The impact of nutrition and lifestyle on male fertility. Arch Ital Urol Androl.

[5] Salas-Huetos A, Bulló M, Salas-Salvadó J (2017). Dietary patterns, foods and nutrients in male fertility parameters and fecundability: a systematic review of observational studies. Hum Reprod Update.

[6] Service CA, Puri D, Al Azzawi S (2023). The impact of obesity and metabolic health on male fertility: a systematic review. Fertil Steril.

[7] Clemente-Suárez VJ, Beltrán-Velasco AI, Redondo-Flórez L (2023). Global Impacts of Western Diet and Its Effects on Metabolism and Health: A Narrative Review. Nutrients.

[8] Ferramosca A, Zara V (2022). Diet and Male Fertility: The Impact of Nutrients and Antioxidants on Sperm Energetic Metabolism. Int J Mol Sci.

[9] Shen CY, Lu CH, Wu CH (2020). The Development of Maillard Reaction, and Advanced Glycation End Product (AGE)-Receptor for AGE (RAGE) Signaling Inhibitors as Novel Therapeutic Strategies for Patients with AGE-Related Diseases. Molecules.

[10] Gill V, Kumar V, Singh K (2019). Advanced Glycation End Products (AGEs) May Be a Striking Link Between Modern Diet and Health. Biomolecules.

[11] Liang Z, Chen X, Li L (2020). The fate of dietary advanced glycation end products in the body: from oral intake to excretion. Crit Rev Food Sci Nutr.

[12] Ma Y, Wang X, Lin S (2025). The Potential Role of Advanced Glycation End Products in the Development of Kidney Disease. Nutrients.

[13] Raghavan CT (2024). Advanced Glycation End Products in Neurodegenerative Diseases. J Mol Neurosci.

[14] Zgutka K, Tkacz M, Tomasiak P (2023). A Role for Advanced Glycation End Products in Molecular Ageing. Int J Mol Sci.

[15] Browning J, Ghanim M, Jagoe W (2024). Membrane-bound receptor for advanced glycation end products (RAGE) is a stable biomarker of low-quality sperm. Hum Reprod Open.

[16] Bagheri V, Hassanshahi G, Zeinali M (2016). Elevated levels of S100A12 in the seminal plasma of infertile men with varicocele. Int Urol Nephrol.

[17] Charalampidou S, Simitsopoulou Μ, Skoura L (2017). Soluble receptor for advanced glycation end products in male infertility. Hippokratia.

[18] Wu S, Yan M, Ge R (2020). Crosstalk between Sertoli and Germ Cells in Male Fertility. Trends Mol Med.

[19] O'Donnell L, Smith LB, Rebourcet D (2022). Sertoli cells as key drivers of testis function. Semin Cell Dev Biol.

[20] Ge X, He Z, Cao C (2022). Protein palmitoylation-mediated palmitic acid sensing causes blood-testis barrier damage via inducing ER stress. Redox Biol.

[21] Kamińska A, Pardyak L, Lustofin S (2024). 9-cis-retinoic acid signaling in Sertoli cells regulates their immunomodulatory function to control lymphocyte physiology and Treg differentiation. Reprod Biol Endocrinol.

[22] Peng YJ, Tang XT, Shu HS (2023). Sertoli cells are the source of stem cell factor for spermatogenesis. Development.

[23] Zhao L, Yao C, Xing X (2020). Single-cell analysis of developing and azoospermia human testicles reveals central role of Sertoli cells. Nat Commun.

[24] Wu D, Zhang K, Khan FA (2022). Smtnl2 regulates apoptotic germ cell clearance and lactate metabolism in mouse Sertoli cells. Mol Cell Endocrinol.

[25] Wang S, Qian Z, Ge X (2022). LncRNA Tug1 maintains blood-testis barrier integrity by modulating Ccl2 expression in high-fat diet mice. Cell Mol Life Sci.

[26] Chen MC, Lin JA, Lin HT (2019). Potential effect of advanced glycation end products (AGEs) on spermatogenesis and sperm quality in rodents. Food Funct.

[27] Akbarian F, Rahmani M, Tavalaee M (2021). Effect of Different High-Fat and Advanced Glycation End-Products Diets in Obesity and Diabetes-Prone C57BL/6 Mice on Sperm Function. Int J Fertil Steril.

[28] Rindone GM, Dasso ME, Centola CL (2023). Sertoli cell adaptation to glucose deprivation: Potential role of AMPK in the regulation of lipid metabolism. J Cell Biochem.

[29] Barazzuol L, Giamogante F, Calì T (2021). Mitochondria Associated Membranes (MAMs): Architecture and physiopathological role. Cell Calcium.

[30] Chen X, Yang Y, Zhou Z (2024). Unraveling the complex interplay between Mitochondria-Associated Membranes (MAMs) and cardiovascular Inflammation: Molecular mechanisms and therapeutic implications. Int Immunopharmacol.

[31] Cao Y, Chen Z, Hu J (2021). Mfn2 Regulates High Glucose-Induced MAMs Dysfunction and Apoptosis in Podocytes via PERK Pathway. Front Cell Dev Biol.

[32] Jiménez R, Zúñiga-Muñoz A, Álvarez-León E (2024). Quercetin preserves mitochondria-endoplasmic reticulum contact sites improving mitochondrial dynamics in aged myocardial cells. Biogerontology.

[33] Allen RJ, Kronemberger A, Shi Q Altered Relaxation and Mitochondria-Endoplasmic Reticulum Contacts Precede Major (Mal)Adaptations in Aging Skeletal Muscle and Are Prevented by Exercise. Aging Cell. 2025: e70137.

[34] Zhang Y, Ma P, Wang S (2025). Restoring calcium crosstalk between ER and mitochondria promotes intestinal stem cell rejuvenation through autophagy in aged Drosophila. Nat Commun.

[35] Puebla-Huerta A, Huerta H, Quezada-Gutierez C (2025). Calcium (Ca(2+)) fluxes at mitochondria-ER contact sites (MERCS) are a new target of senolysis in therapy-induced senescence (TIS). NPJ Aging.

[36] Khalid M, Petroianu G, Adem A (2022). Advanced Glycation End Products and Diabetes Mellitus: Mechanisms and Perspectives. Biomolecules.

[37] Castellini C, Mattioli S, Signorini C (2019). Effect of Dietary n-3 Source on Rabbit Male Reproduction. Oxid Med Cell Longev.

[38] Safarinejad MR (2011). Effect of omega-3 polyunsaturated fatty acid supplementation on semen profile and enzymatic anti-oxidant capacity of seminal plasma in infertile men with idiopathic oligoasthenoteratospermia: a double-blind, placebo-controlled, randomised study. Andrologia.

[39] Koppers M, Özkan N, Farías GG (2020). Complex Interactions Between Membrane-Bound Organelles, Biomolecular Condensates and the Cytoskeleton. Front Cell Dev Biol.

[40] Xian T, Liu Y, Ye Y (2024). Human salivary histatin 1 regulating IP3R1/GRP75/VDAC1 mediated mitochondrial-associated endoplasmic reticulum membranes (MAMs) inhibits cell senescence for diabetic wound repair. Free Radic Biol Med.

[41] Frankhouser DE, Steck S, Sovic MG (2022). Dietary omega-3 fatty acid intake impacts peripheral blood DNA methylation -anti-inflammatory effects and individual variability in a pilot study. J Nutr Biochem.

[42] Liang K, Yao L, Wang S (2021). miR-125a-5p increases cellular DNA damage of aging males and perturbs stage-specific embryo development via Rbm38-p53 signaling. Aging Cell.

[43] Sourris KC, Harcourt BE, Penfold SA (2010). Modulation of the cellular expression of circulating advanced glycation end-product receptors in type 2 diabetic nephropathy. Exp Diabetes Res.

[44] Ge X, Pan P, Jing J (2018). Rosiglitazone ameliorates palmitic acid-induced cytotoxicity in TM4 Sertoli cells. Reprod Biol Endocrinol.

[45] Jing J, Ouyang L, Zhang H (2024). Omega-3 polyunsaturated fatty acids and its metabolite 12-HEPE rescue busulfan disrupted spermatogenesis via target to GPR120. Cell Prolif.

[46] Li J, Tan J, Wang T (2024). cGAS-ISG15-RAGE axis reprogram necroptotic microenvironment and promote lymphatic metastasis in head and neck cancer. Exp Hematol Oncol.

[47] Wang B, Jiang T, Qi Y (2024). AGE-RAGE Axis and Cardiovascular Diseases: Pathophysiologic Mechanisms and Prospects for Clinical Applications. Cardiovasc Drugs Ther.

[48] Zheng DL, Wu QR, Zeng P (2022). Advanced glycation end products induce senescence of atrial myocytes and increase susceptibility of atrial fibrillation in diabetic mice. Aging Cell.

[49] Simon L, Ekman GC, Garcia T (2010). ETV5 regulates sertoli cell chemokines involved in mouse stem/progenitor spermatogonia maintenance. Stem Cells.

[50] Chen SR, Liu YX (2015). Regulation of spermatogonial stem cell self-renewal and spermatocyte meiosis by Sertoli cell signaling. Reproduction.

[51] Du H, Ma Y, Wang X (2023). Advanced glycation end products induce skeletal muscle atrophy and insulin resistance via activating ROS-mediated ER stress PERK/FOXO1 signaling. Am J Physiol Endocrinol Metab.

[52] Suzuki S, Hayashi T, Egawa T (2024). Advanced glycation end products promote ROS production via PKC/p47 phox axis in skeletal muscle cells. J Physiol Sci.

[53] Zhang R, Jiang L, Li G (2022). Advanced Glycosylation End Products Induced Synaptic Deficits and Cognitive Decline Through ROS-JNK-p53/miR-34c/SYT1 Axis in Diabetic Encephalopathy. J Alzheimers Dis.

[54] Andreone L, Ambao V, Pellizzari EH (2017). Role of FSH glycan structure in the regulation of Sertoli cell inhibin production. Reproduction.

[55] Li L, Gao Y, Chen H (2017). Cell polarity, cell adhesion, and spermatogenesis: role of cytoskeletons. F1000Res.

[56] Huang K, Ru B, Zhang Y (2019). Sertoli cell-specific coxsackievirus and adenovirus receptor regulates cell adhesion and gene transcription via β-catenin inactivation and Cdc42 activation. Faseb j.

[57] Liu L, Zhang Y, Chang X (2018). Fluorochloridone perturbs blood-testis barrier/Sertoli cell barrier function through Arp3-mediated F-actin disruption. Toxicol Lett.

[58] Sakamoto S, Thumkeo D, Ohta H (2018). mDia1/3 generate cortical F-actin meshwork in Sertoli cells that is continuous with contractile F-actin bundles and indispensable for spermatogenesis and male fertility. PLoS Biol.

[59] Lu A, Xu Z, Zhao Z (2024). Double Braking Effects of Nanomedicine on Mitochondrial Permeability Transition Pore for Treating Idiopathic Pulmonary Fibrosis. Adv Sci (Weinh).

[60] Arruda AP, Pers BM, Parlakgül G (2014). Chronic enrichment of hepatic endoplasmic reticulum-mitochondria contact leads to mitochondrial dysfunction in obesity. Nat Med.

[61] Wang Z, Du X, Yu S (2024). Mitochondria-Associated Membranes in Aging and Senescence. Aging Dis.

[62] Li Z, Hu O, Xu S (2024). The SIRT3-ATAD3A axis regulates MAM dynamics and mitochondrial calcium homeostasis in cardiac hypertrophy. Int J Biol Sci.

[63] Yao S, Wei X, Deng W (2022). Nestin-dependent mitochondria-ER contacts define stem Leydig cell differentiation to attenuate male reproductive ageing. Nat Commun.

[64] Housmans JAJ, Wu G, Schymkowitz J (2023). A guide to studying protein aggregation. Febs j.

[65] Deepu V, Rai V, Agrawal DK (2024). Quantitative Assessment of Intracellular Effectors and Cellular Response in RAGE Activation. Arch Intern Med Res.

[66] Tully CA, Alesi S, McPherson NO (2024). Assessing the influence of preconception diet on male fertility: a systematic scoping review. Hum Reprod Update.

[67] Karimi J, Goodarzi MT, Tavilani H (2012). Increased receptor for advanced glycation end products in spermatozoa of diabetic men and its association with sperm nuclear DNA fragmentation. Andrologia.

[68] Mallidis C, Agbaje IM, Rogers DA (2009). Advanced glycation end products accumulate in the reproductive tract of men with diabetes. Int J Androl.

[69] Cepas V, Collino M, Mayo JC (2020). Redox Signaling and Advanced Glycation Endproducts (AGEs) in Diet-Related Diseases. Antioxidants (Basel).

[70] Roncero-Ramos I, Niquet-Léridon C, Strauch C (2014). An advanced glycation end product (AGE)-rich diet promotes Nε-carboxymethyl-lysine accumulation in the cardiac tissue and tendons of rats. J Agric Food Chem.

[71] El-Kamshoushi AM, Zohdy NI, Abou Khedr NA (2013). Ultrastructure of the seminiferous tubules in oligoasthenoteratozoospermic men associated with varicocele. Andrologia.

[72] Darmishonnejad Z, Zadeh VH, Tavalaee M (2024). Effect of Advanced Glycation end Products (AGEs) on Sperm Parameters and Function in C57Bl/6 Mice. Reprod Sci.

[73] Zhao YT, Qi YW, Hu CY (2016). Advanced glycation end products inhibit testosterone secretion by rat Leydig cells by inducing oxidative stress and endoplasmic reticulum stress. Int J Mol Med.

[74] Dong S, Chen C, Zhang J (2022). Testicular aging, male fertility and beyond. Front Endocrinol (Lausanne).

[75] Deng Z, Zhao L, Li S (2024). Targeting dysregulated phago-/auto-lysosomes in Sertoli cells to ameliorate late-onset hypogonadism. Nat Aging.

[76] López-Otín C, Blasco MA, Partridge L (2023). Hallmarks of aging: An expanding universe. Cell.

[77] Davalli P, Mitic T, Caporali A (2016). ROS, Cell Senescence, and Novel Molecular Mechanisms in Aging and Age-Related Diseases. Oxid Med Cell Longev.

[78] Li X, Li C, Zhang W (2023). Inflammation and aging: signaling pathways and intervention therapies. Signal Transduct Target Ther.

[79] Huang CY, Ng MY, Lin T (2024). Quercetin ameliorates advanced glycation end product-induced wound healing impairment and inflammaging in human gingival fibroblasts. J Dent Sci.

[80] Victorelli S, Salmonowicz H, Chapman J (2023). Apoptotic stress causes mtDNA release during senescence and drives the SASP. Nature.

[81] Xi H, Shan W, Li M (2024). Trehalose attenuates testicular aging by activating autophagy and improving mitochondrial quality. Andrology.

[82] Kongmanas K, Saewu A, Kiattiburut W (2021). Accumulation of Seminolipid in Sertoli Cells Is Associated with Increased Levels of Reactive Oxygen Species and Male Subfertility: Studies in Aging Arsa Null Male Mice. Antioxidants (Basel).

[83] Chandrasekaran A, Idelchik M, Melendez JA (2017). Redox control of senescence and age-related disease. Redox Biol.

[84] Song J, Gu L, Ren X (2020). Prediction model for clinical pregnancy for ICSI after surgical sperm retrieval in different types of azoospermia. Hum Reprod.

[85] Dan Dunn J, Alvarez LA, Zhang X (2015). Reactive oxygen species and mitochondria: A nexus of cellular homeostasis. Redox Biol.

[86] Zhang P, Yan X, Zhang X (2023). TMEM215 Prevents Endothelial Cell Apoptosis in Vessel Regression by Blunting BIK-Regulated ER-to-Mitochondrial Ca Influx. Circ Res.

[87] Zeeshan HM, Lee GH, Kim HR (2016). Endoplasmic Reticulum Stress and Associated ROS. Int J Mol Sci.

[88] Wang X, Wen Y, Dong J (2018). Systematic In-Depth Proteomic Analysis of Mitochondria-Associated Endoplasmic Reticulum Membranes in Mouse and Human Testes. Proteomics.

[89] Guo X, Wang L, Xuan J (2025). Fluoride induces spermatocyte apoptosis by IP3R1/MCU-mediated mitochondrial calcium overload through MAMs. J Hazard Mater.

[90] Giacomello M, Pellegrini L (2016). The coming of age of the mitochondria-ER contact: a matter of thickness. Cell Death Differ.

[91] Krebs J, Agellon LB, Michalak M (2015). Ca(2+) homeostasis and endoplasmic reticulum (ER) stress: An integrated view of calcium signaling. Biochem Biophys Res Commun.

[92] Wise J (1997). Diabetes drug withdrawn after reports of hepatic events. Bmj.

[93] Rosso A, Pansera M, Zamoner A (2012). 1α,25(OH)2-Vitamin D3 stimulates rapid plasma membrane calcium influx via MAPK activation in immature rat Sertoli cells. Biochimie.

[94] Zhang A, Williamson CD, Wong DS (2011). Quantitative proteomic analyses of human cytomegalovirus-induced restructuring of endoplasmic reticulum-mitochondrial contacts at late times of infection. Mol Cell Proteomics.

[95] Poston CN, Krishnan SC, Bazemore-Walker CR (2013). In-depth proteomic analysis of mammalian mitochondria-associated membranes (MAM). J Proteomics.

[96] Veitia RA, Govindaraju DR, Bottani S (2017). Aging: Somatic Mutations, Epigenetic Drift and Gene Dosage Imbalance. Trends Cell Biol.

[97] Sherratt SCR, Juliano RA, Copland C (2021). EPA and DHA containing phospholipids have contrasting effects on membrane structure. J Lipid Res.

[98] Capece U, Gugliandolo S, Morciano C (2024). Erythrocyte Membrane Fluidity and Omega-3 Fatty Acid Intake: Current Outlook and Perspectives for a Novel, Nutritionally Modifiable Cardiovascular Risk Factor. Nutrients.

