# Supplementary material for: The Remodeling of Mitochondrial-Endoplasmic Reticulum Contacts by Omega-3 Fatty Acids Mitigates Dietary Advanced Glycation End Product-Driven Sertoli Cell Senescence and Oligoasthenozoospermia
Source: Int J Biol Sci. 2025 Sep 27;21(14):6042–61. doi: 10.7150/ijbs.117091 (PMC12594550; doi:10.7150/ijbs.117091)
Supplement: Supplementary file 1 — Supplementary methods, figures and tables. [file ijbsv21p6042s1.pdf]

Supplementary Materials

Table S1. Summary of the Basic characteristics and semen parameters of participants.

No.	Age	Sperm concentration (10^6/mL)	Semen volume (mL)	Total motility (%)	Progressive motility (%)	Normal morphology (%)	FSH (IU/L)	LH (IU/L)	T (nmol/L)	Inhibin B (pg/mL)	Type	Serum AGEs (μg/ml)
1	25	2.67	0.6	57.14	57.14	2.29	5.73	6.26	14.04	107.96	OAZ	9.10
2	35	9.02	2	26.17	12.15	3.18	10.73	4.44	15.32	51.45	OAZ	7.63
3	28	10.62	8	29.06	10.62	4.59	5.43	2.18	11.34	132.44	OAZ	4.26
4	32	7.12	3.8	46.46	41.94	1.79	4.57	7.65	8.75	65.82	OAZ	5.40
5	30	2.22	2.5	80	80	4.56	20.56	10.83	12.36	21.61	OAZ	7.32
6	33	8.55	3.9	67.53	59.74	5.61	7.26	7.14	2.38	85.88	OAZ	6.36
7	27	5.73	2.8	55.88	51.47	4.09	1.95	2.03	10.56	55.44	OAZ	6.14
8	33	0.51	4	50	50	2.33	17.91	8.26	8.74	24.12	OAZ	8.41
9	27	7.29	4	30.69	22.77	4.21	6.48	6.91	14.23	150.48	OAZ	10.27
10	29	2.44	4	20.68	10.34	3.45	4.8	3.29	22.39	151.81	OAZ	4.52
11	36	9.02	3.5	42.05	30.84	5.61	4.6	4.76	26.75	99.19	OAZ	13.84
12	38	2.35	3.7	55.91	51.61	5.45	4.6	4.76	26.75	99.19	OAZ	6.95
13	33	111.35	1	32.35	24.18	4.63	2.82	3.24	21.25	150.37	OAZ	6.61
14	30	5.87	3	30.3	24.24	1.83	12.28	8.57	15.33	64.11	OAZ	5.36
15	29	172.4	1.2	36.95	26.98	4.69	6.07	2.66	5.35	97.93	OAZ	6.69
16	23	50.05	1.8	24.14	18.18	3.59	3.16	2.9	13.81	142.51	OAZ	5.09
17	26	3.67	3.3	30.3	21.21	4.17	8.98	17.41	9.08	17.23	OAZ	5.19
18	44	12.44	7.2	0.5	0.5	4.17	2.52	2.83	8.59	124.22	OAZ	4.26
19	31	158.82	3.3	42.81	30.03	4.13	6.34	6.41	18.27	140.28	OAZ	4.10
20	43	166.84	3.5	39.7	30.61	5.19	11.44	9.56	18.35	81.75	OAZ	6.91
21	36	143.08	2.1	27.56	14.13	4.67	3.36	2.28	7.51	119.53	OAZ	4.26
22	37	132.46	4.2	37.41	29.01	4.15	3.96	6	17.69	126.22	OAZ	3.91
23	31	174.42	4	4.35	1.74	4.72	4.05	5.04	12.37	85.06	OAZ	3.65
24	27	1.11	4.9	20	20	1.35	2.86	2.91	9.17	66.98	OAZ	4.79
25	26	157.23	2.9	75.24	62.7	5.58	7.76	8.07	15.15	118.92	CTL	4.97
26	36	193.63	1	64.49	50.65	5.5	6.93	5.02	22.51	105.19	CTL	4.52
27	31	64.71	5	49.22	41.02	5.14	6.94	4.26	24.96	98.91	CTL	4.61
28	32	351.37	3	68.92	45.47	4.17	4.99	3.51	19.23	154.47	CTL	4.79
29	27	48.37	3.4	48.08	41.81	4.15	4.04	7.24	11.63	88.09	CTL	7.43
30	31	127.78	2.4	72.7	61.42	4.69	2.87	2.53	19.58	144.93	CTL	4.97
31	28	163.3	3.9	60.99	50.15	4.17	8.23	4.65	19.15	111.79	CTL	4.74
32	35	93.53	1.5	62.97	53.24	4.17	8.11	4.41	13.79	79.91	CTL	3.64
33	30	215.88	4	83.84	73.3	5.69	4.14	2.16	14.56	164.15	CTL	4.21
34	29	60.16	3.1	62.18	50	4.23	15.78	15.23	15.61	101.66	CTL	6.15
35	28	51.57	3.3	52.62	44.12	4.69	2.59	3.29	10.29	198.54	CTL	3.81
36	38	242.17	3.5	73.07	54.07	4.67	5.9	4.9	16.44	145.86	CTL	3.77
37	26	121.84	4	44.81	33.61	4.19	3.91	2.86	8.06	152.32	CTL	3.18
38	30	342.27	6	72.08	54.21	5.14	4.83	2.3	14.06	147.28	CTL	3.81
39	39	112.74	2.4	66.37	50.67	6.1	5.89	3.41	12.94	90.3	CTL	7.68
40	24	91.85	4.8	57.34	55.05	4.67	3.05	2.71	10.28	204.43	CTL	3.51
41	27	112.74	4.6	56.5	44.39	4.65	6.26	4.92	16.09	123.78	CTL	4.34
42	30	161.28	1.8	81.81	73.35	5.21	4.32	3.05	17.58	139.76	CTL	3.47
43	31	148.39	2	69.33	54	5.16	4.63	3.27	19.35	114.69	CTL	3.47
44	27	56.12	5.6	83.33	71.62	5.14	3.67	4.09	19.51	99.15	CTL	4.79
45	30	41.29	2.4	68.57	56.72	4.69	5.86	6.96	35.25	145.62	CTL	4.79
46	28	43.82	5	41.92	32.69	4.65	3.59	5.66	22.31	122.66	CTL	5.11
47	31	259.86	4.4	55.25	38.91	5.16	5.4	5.11	19.88	109.11	CTL	6.10
48	29	78.64	4	49.2	36.98	4.17	5.90	4.20	13.28	112.92	CTL	4.52

Table S2. Basic characteristics and semen parameters of CTL and OAZ participants.

Parameters	CTL (N=24)	OAZ (N=24)	Total (N=48)	*p* -value
Age	30.125±3.666	31.792±5.220	30.958±4.587	0.217
Concentration (10^6/mL)	139.189±87.767	50.002±66.719	94.596±89.810	<0.001
Semen volume (ml)	3.500±1.285	3.429±1.664	3.465±1.487	0.872
Progressive motility (%)	51.256±11.249	29.976±19.408	40.616±19.100	<0.001
Total Motility(%)	63.368±11.851	36.998±17.988	50.183±20.146	<0.001
DFI (%)	19.193±11.655	29.405±13.529	24.299±13.620	0.009
HDS (%)	8.143±2.555	10.053±4.842	9.098±3.987	0.101
Normal morphology(%)	4.824±0.546	3.935±1.203	4.380±1.034	0.003
FSH (IU/L)	5.650±2.642	6.769±4.660	6.259±3.877	0.275
LH (IU/L)	4.742±2.662	5.763±3.462	5.263±3.166	0.254
T (nmol/L)	17.145±5.597	13.766±6.156	15.877±5.860	0.131
INHB (ng/L)	128.102±31.764	94.233±41.118	111.167±40.455	0.003
Serum AGEs (μg/ml)	4.683±1.145	6.291±2.315	5.487±1.996	0.005

The results are presented as the mean ± SD, p-value got from t-test

Table S3. Univariate and multivariate Logistic Regression Analysis of potential risk factors for OAZ.

Variables	Univariate					Multivariate				
	β	S.E	Z	P	OR (95%CI)	β	S.E	Z	P	OR (95%CI)
**Serum AGEs**	0.62	0.24	2.54	0.011	1.86 (1.15 ~ 3.00)	0.52	0.26	2.01	0.044	1.68 (1.01 ~ 2.79)
**FSH**	0.08	0.08	0.99	0.324	1.08 (0.92 ~ 1.27)	-0.13	0.15	-0.84	0.399	0.88 (0.65 ~ 1.18)
**LH**	0.11	0.1	1.09	0.274	1.12 (0.92 ~ 1.37)	0.02	0.18	0.12	0.905	1.02 (0.72 ~ 1.45)
**T**	-0.1	0.05	-1.83	0.067	0.90 (0.81 ~ 1.01)	-0.13	0.07	-1.7	0.089	0.88 (0.76 ~ 1.02)
**INHB**	-0.03	0.01	-2.63	0.009	0.97 (0.96 ~ 0.99)	-0.02	0.01	-1.44	0.15	0.98 (0.96 ~ 1.01)
**Age**	0.08	0.07	1.23	0.217	1.09 (0.95 ~ 1.24)	0.09	0.08	1.08	0.28	1.10 (0.93 ~ 1.29)

OR: Odds Ratio, CI: Confidence Interval

Table S4. The antibodies used in the experiments.

Antibody name	Manufacture (catalogue number)	Applications (working dilution)
RAGE	ABclonal (A13264)	WB (1:5000)
P21	ProteinTech (28248-1-AP)	IHC (1:100)
P16	Abcam(ab108349)	IHC (1:100)
c-Kit	Abcam(ab32363)	IHC (1:400)
SYCP3	Abcam(ab97672)	IF(1:4000)
SOX9	Sigma-Aldrich (AB5535)	WB (1:5000) IHC (1:500)
ZO-1	ABclonal (A11417)	WB (1:1000)
Occludin	ProteinTech (13409-1-AP)	WB (1:3000)
Vinculin	ProteinTech (66305-1-Ig)	WB (1:5000)
CLDN5	ABclonal (A10207)	WB (1:1000)
CLDN11	ABclonal (A12478)	WB (1:1000)
BIP	Abcam (ab21685)	WB (1:1000)
CHOP	ProteinTech (15204-1-AP)	WB (1:1000)
ATF4	ABclonal (A18687)	WB (1:1000)
ATF6	ABclonal (A0202)	WB (1:1000)
VDAC1	Abcam(ab14734)	WB (1:1000) IF(1:200)
IP3R1	Abcam(ab5804)	WB (1:1000) IF(1:100)
MCU	CST(#14997)	WB (1:1000)
Goat anti-Mouse IgG (H+L) Secondary Antibody, HRP	Invitrogen (31430)	WB (1:5000)
Goat anti-Rabbit IgG (H+L) Secondary Antibody, HRP	Invitrogen (31460)	WB (1:5000)

Table S5. The primers used in the experiments.

Gene	Forward (5'-3')	Reverse (5'-3')
rage	CCACTGGAATTGTCGATGAGG	CTCGGACTCGGTAGTTGGACT
zo-1	AGCGAATGTCTAAACCTGGG	TCCAACTTGAGCATACACAGG
occludin	GGCAAGCGATCATACCCAGA	GCTGCCTGAAGTCATCCACA
cxcl12	TGCATCAGTGACGGTAAAC	CAGCCGTGCAACAATCT
etv5	AGGCTCTTGGTGCTAAGTATG	AATAGGGAATTCTGATGGGTGG
fgf2	TCTACTGCAAGAACGGCG	CTCCCTTGATAGACACAACTCC
gdnf	AGAGGGGCAAAAATCGGGG	CCGCTGCAATATCGAAAGATCA
InhβB	TCCGAGATCATCAGCTTTGC	GGGAGCAGTTTCAGGTACAG
Inhα	GCACAGGACCTCTGAACCAG	GGGATGGCCGGAATACATAAG
star	ATGTTCCTCGCTACGTTCAAG	CCCAGTGCTCTCCAGTTGAG
sox9	GAGCCGGATCTGAAGAGGGA	GCTTGACGTGTGGCTTGTTC
wt1	ATGACCTCCCAGCTTGAATG	GTTCTCACTCTCATACCCTGTG
claudin5	GCAAGGTGTATGAATCTGTGCT	GTCAAGGTAACAAAGAGTGCCA
claudin11	AAATGGACGAACTGGGCTC	AATCATGAGGGCTCTACAAGC
lin28	GGCATCTGTAAGTGGTTCAACG	GCCAGTGACACGGATGGATT
stra8	ACAACCTAAGGAAGGCAGTTTAC	GACCTCCTCTAAGCTGTTGGG
sycp3	AGACATGGGACATGAAGTAGGC	CCTCTCTCGTTCGTTCGTTTT
acrv1	TCAGCAACTTTCAAGCGAGTAT	CTCCTGAAGAGTGCTCACCTG
prm1	TCCGACTCATGTTGAAAAACCC	CCTTCCCCGGATTCCTGTCT
icam1	GAGCCAATTTCTCATGCCGC	TCGAGCTTTGGGATGGTAGC
icam2	GCATCCTCAAGGGAAGTGGA	TTCACCCACAAACACCCGAG
nectin2	TCCAGATTGTCACCGACGC	CACTCGTACCCGCACATCTT
nectin3	GCAAAGCCGTTACATTCCCA	CCCAGTCAATCTGTGCGACT
N-cadherin	CACATCAGGACACCCAACGG	GAACGTCCCGAGCAAGGAT
α-cadherin	AAGCAGGCTCTCGAATGGAC	CTGAACCTCGGCCTTCACTT
β-catenin	CACATCAGGACACCCAACGG	GAACGTCCCGAGCAAGGAT
ccl5	GCCCACGTCAAGGAGTATTTCT	ACAAACACGACTGCAAGATTGG
ccl7	CCACATGCTGCTATGTCAAGA	ACACCGACTACTGGTGATCCT
cxcl1	ACTGCACCCAAACCGAAGTC	TGGGGACACCTTTTAGCATCTT
ifn-β	AGCTCCAAGAAAGGACGAACA	GCCCTGTAGGTGAGGTTGAT
ifn-γ	GCCACGGCACAGTCATTGA	TGCTGATGGCCTGATTGTCTT
il-10	CTTACTGACTGGCATGAGGATCA	GCAGCTCTAGGAGCATGTGG
il-17A	GGACTCTCCACCGCAATGAA	GCACTGAGCTTCCCAGATCAC
il-1α	TGCAGTCCATAACCCATGATC	ACAAACTTCTGCCTGACGAG
il-1β	GCCATCCTCTGGACTCAT	AGGCCACAGGTATTTTGTCG
il-6	CTACCAAACTGGATATAATCAGGA	CCAGGTAGCTATGGTACTCCAGAA
tnf-α	ATGAGCACAGAAAGCATGATC	TACAGGCTTGTCACTCGAATT
ip3r1	CGTTTTGAGTTTGAAGGCGTTT	CATCTTGCGCCAATTCCCG
vdac1	CCCACATACGCCGATCTTGG	GTGGTTTCCGTGTTGGCAGA
mfn2	GTGGGCTGGAGACTCATCG	CTCACTGGCGTATTCCGCAA
ptpip51	TGGTGGCTGGGTACACTATGT	GCTGGCTGTAAAGGACGCA
slc25a46	CCGAGCAGCGAACAGTTGAA	CGGCGTAAAACAATGCAAGGA
drp1	CAGGAATTGTTACGGTTCCCTAA	CCTGAATTAACTTGTCCCGTGA
atad3a	CAAATGGAGCAACTTCGACCC	GGCAGCTTCGTACTCCTTGA
mcu	GGTCCAGCAACTATACACCACA	CAAAGTGGTCCTCTTCTCAGCT
m36b4	GAAACTGCTGCCTCACATCCG	GCTGGCACAGTGACCTCACACG

Supplementary Figures

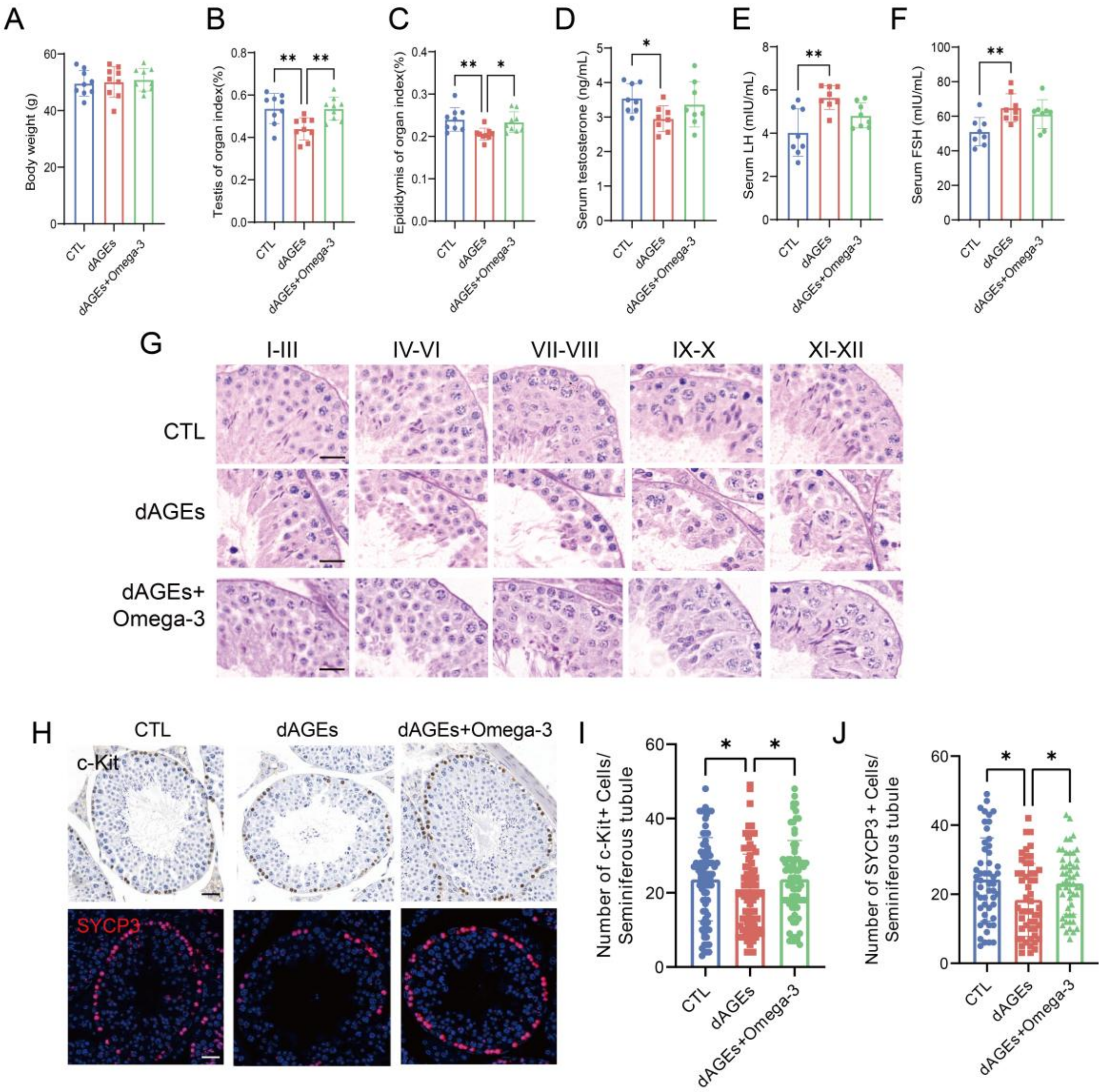

Fig. S1 Basic indexes of mice treated with AGE-rich diet with or without Omega-3.

All animals were randomly divided into three groups: a control group (CTL), an advanced glycation end product-rich diet group (dAGEs) for 16 weeks, and a group fed an AGE-rich diet supplemented with omega-3 PUFAs for the last 4 weeks (dAGEs + Omega-3). (A-C) Body weight(A), organ index for the testis (B) and epididymis (C) of the mice after 16-week treatment. (D-F) Concentrations of hormone in serum including testosterone (T) (D), LH (E) and FSH (F). (G) Representative images of testicular sections from modeled mice stained with PAS and grouped according to the stages of seminiferous tubules. Scale bar: 50 μm. (H-J) Immunohistochemistry staining for c-Kit, a marker for undifferentiated spermatogonia, and immunofluorescence staining for SYCP3, a marker for spermatocytes. Scale bar = 50μm. Statistical analysis of the number of c-Kit+ cells or SYCP3+ cells in each seminiferous tubule across different groups. Statistical analysis between multiple groups was performed by one-way ANOVA. A two-sided p-value < 0.05 was considered to be statistically significant. The level of significance defined as $p < 0.05$ (*), $p < 0.01$ (**).

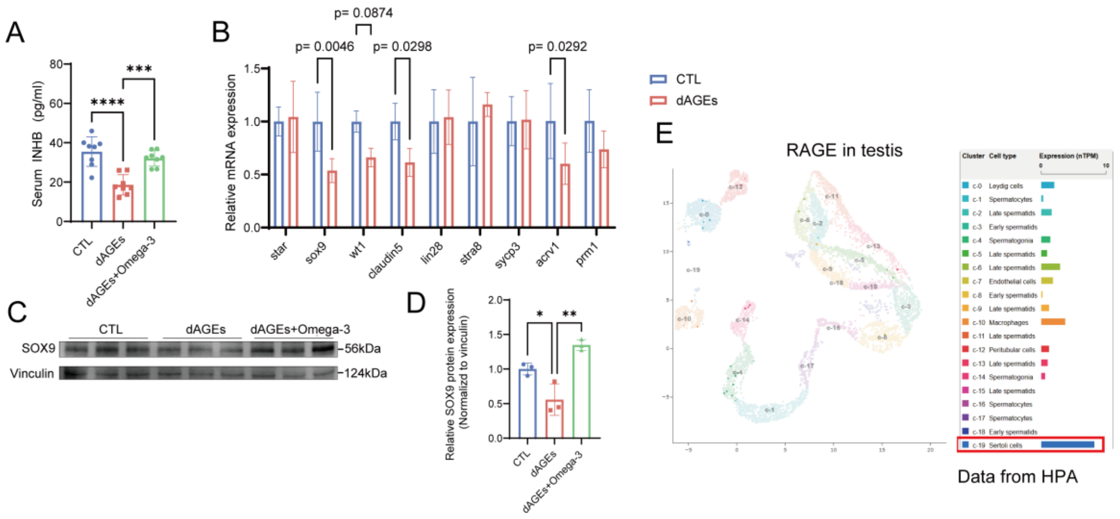

Fig. S2 AGEs exert effects on Sertoli cells via the RAGE.

(A) Concentrations of INHB in serum of mice. (B)The mRNA levels of various cellular markers in the testicular microenvironment of mice testis in the CTL and dAGEs groups, including Leydig cell (star), Sertoli cell (sox9, wt1, claudin9) Spermatogonial Stem Cell (lin28) Spermatogonia (stra8) Spermatocyte (sycp3) Round spermatid (acrv1) Elongated spermatid (prm1). (C, D) The protein expression of SOX9 in mice testis was detected using Western blotting and quantified by ImageJ and normalized to Vinculin level. (E) Cell clustering analysis reveals the various cell types present in the testis, and a bar chart displays the expression levels of RAGE across these cell types. The single-cell data is derived from the Human Protein Atlas (https://www.proteinatlas.org). (F) Quantification of the percentage of adherent germ cells change fold to CTL group. Statistical analysis between two groups was performed using unpaired t-test. Statistical analysis between multiple groups was performed by one-way ANOVA. A two-sided p-value < 0.05 was considered to be statistically significant. The level of significance defined as p < 0.05 (*), p < 0.01 (**).

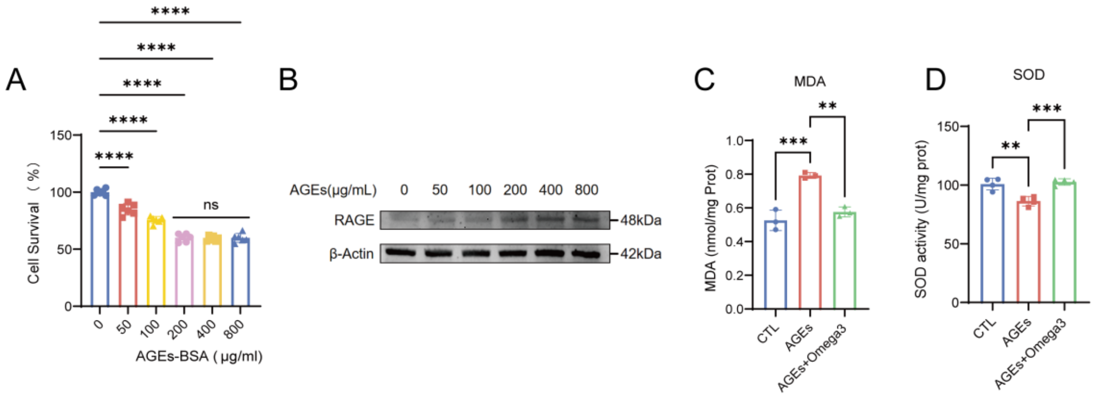

Fig. S3 Omega-3 alleviates oxidative stress imbalance and SASP over-secretion *in vitro* by targeting RAGE.

(A) Analysis of TM4 cells survival following treatment with a gradient of AGEs concentrations (0, 50, 100, 200, 400, 800 μg/mL). (B) The protein expression of RAGE in TM4 cells was detected using Western blotting under a gradient of AGEs concentrations. (C, D) The SOD activity and MDA levels in TM4 cells normalized per mg of protein. Statistical analysis between multiple groups was performed by one-way ANOVA. A two-sided p-value < 0.05 was considered to be statistically significant. The level of significance defined as $p < 0.05$ (*), $p < 0.01$ (**), $p < 0.005$(***), $p < 0.001$ (****).

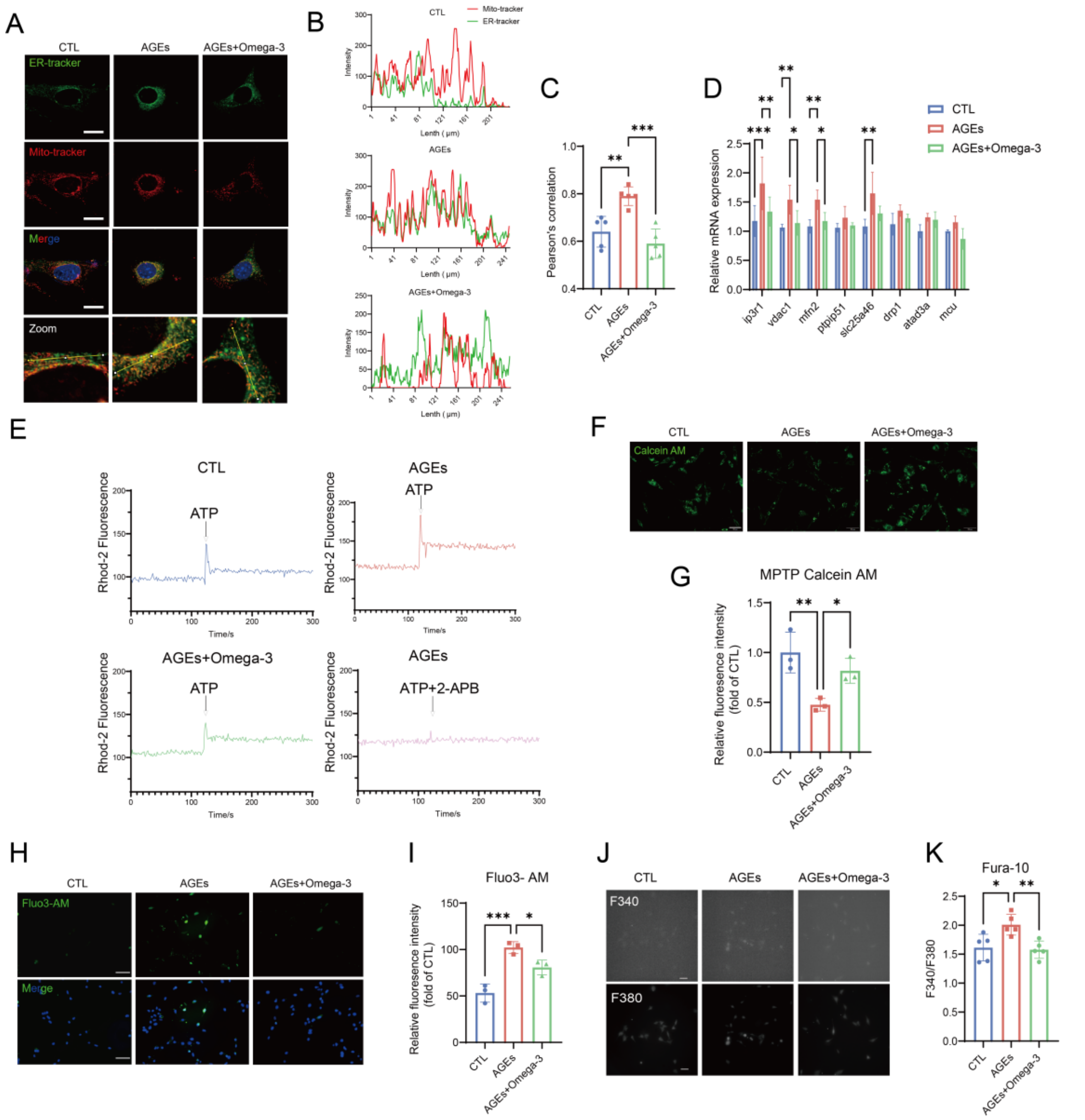

Fig. S4 AGEs treated TM4 cell establish more mitochondria-endoplasmic reticulum contacts and is reduced by omega-3.

(A-C) Representative immunostaining pictures of colocalization between mitochondria and ER in treated TM4 cells(A). Mitochondria and ER are marked with Mito-tracker and ER-tracker. Scale bar: 10μm. Line intensity profile analysis of Mito-tracker and ER-tracker (B). Intensity values were measured along a path indicated in pictures. The Pearson's correlation between ER-tracker and Mito-tracker was analyzed (C). (D) The mRNA levels of MERCs related genes in treated TM4 cells. (E) Rhod-2 fluorescence was then quantified using a microplate reader (Ex/Em = 540/590 nm, cut-off = 570 nm) following stimulation with 10 μM ATP. To assess IP3R function, cells were pretreated with 100 μM 2-APB for 2 min prior to stimulation. Baseline fluorescence (F_0) was defined as the background-subtracted intensity of Rhod-2-loaded cells measured immediately before ATP exposure. (F-G) Representative images of the mitochondrial permeability transition pore (MPTP) by Calcein AM staining in treated TM4 cell and the fluorescence densities were calculated using ImageJ. Scale bar: 50μm. (H-I) Representative images of Cytosolic Ca^{2+} detection using Fluo-3 staining and the fluorescence densities were calculated using ImageJ. Scale bar: 50μm. (J-K)

Representative images depicting intracellular calcium levels in treated TM4 cells, as measured by FURA-10 staining. The fluorescence ratio F340/F380 indicates the baseline calcium levels within these cells. These measurements were calculated using ImageJ software. Scale bar: 50μm. Statistical analysis between multiple groups was performed by one-way ANOVA. A two-sided p-value < 0.05 was considered to be statistically significant. The level of significance defined as $p < 0.05$ (*), $p < 0.01$ (**), $p < 0.005$(***).

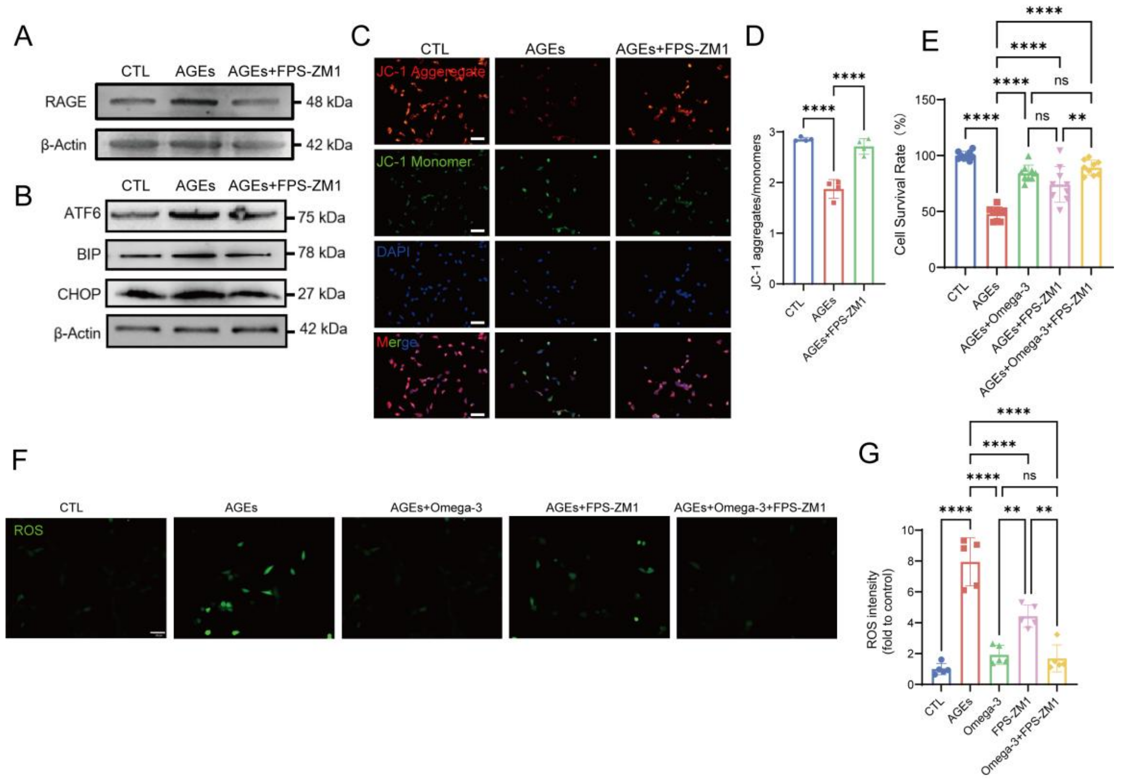

Fig.S5 The RAGE inhibitor FPS-ZM1 mitigates endoplasmic reticulum stress and mitochondrial dysfunction in AGEs-treated TM4 cells.
(A)The protein expression of RAGE in TM4 cells was detected using Western blotting. (B) ER stress protein levels in treated TM4 cells were examined using western blotting. (C, D) Mitochondrial membrane potential (Δψm) in treated TM4 cells was detected using JC-1 staining. (D)Representative images of JC-1 aggregates (red) and JC-1 monomers (green) The ratio of JC-1 aggregates to JC-1 monomers was calculated using ImageJ (D). (E) Quantification of the percentage of adherent germ cells change fold to CTL group. (F) Evaluated the survival of TM4 cells after exposure to AGEs at a concentration of 200 μg/ml, and investigated the protective effects of Omega-3, FPS-ZM1, as well as their combination, using the CCK-8 assay. (G, H) ROS detection using DCFH-DA staining and the fluorescence densities were calculated using ImageJ. Scale bar: 50μm. Statistical analysis between multiple groups was performed by one-way ANOVA. A two-sided p-value < 0.05 was considered to be statistically significant. The level of significance defined as p < 0.001 (****).

Supplementary Materials and Methods

Enzyme-Linked Immunosorbent Assay

Blood samples from mice were centrifuged at 4°C for 10 min at 4,000 rpm, and the serum was collected for hormone detection. Follicle-stimulating hormone (FSH), luteinizing hormone (LH), testosterone (T) and inhibin B (INHB) levels in serum samples were analyzed by ELISA kits (MLBIO, Shanghai, China) according to the manufacturer's instructions. Briefly, standards and serum samples were added into the precoated well and incubated with peroxidase-conjugated anti-Hormones IgG for 1 h. Chromogenic substrates A and B were added and incubated for 15 min at 37°C, and the plate was subjected to reading at 450 nm; their values were calculated according to the standard curve.

Histopathological examinations and PAS staining

The testis and epididymis samples were fixed in Bouin's fixative solution for 24 hours, dehydrated in gradient ethanol, and embedded in paraffin for sectioning into 5 μm slices. These sections were mounted onto glass slides.

For histological staining, the samples underwent H&E staining and PAS staining (Solarbio, China), following the instructions provided by the respective kit manufacturers. For H&E staining, the sections were subjected to dewaxing with xylene and hydration using a gradually decreasing concentration of ethanol. After washing with water for 2 min, the sections were stained with hematoxylin for 5 min, followed by another water wash for 10 min. Subsequently, the sections were stained with eosin solution for 2 min.

Regarding PAS staining, the dewaxed and hydrated slides were treated with periodic acid solution for 10 min, followed by a wash for 5 min with water. Subsequently, the slides were stained with Schiff's reagent and hematoxylin. Finally, all sections were dehydrated, made transparent, and mounted using neutral resin. The slides were then observed under a light microscope, and relevant sections were photographed to facilitate histological observations.

Immunohistochemistry (IHC) and Immunofluorescent staining (IF).

To assessment of immunohistochemical staining, tissue sections were treated with 3% H2O2 to inhibit endogenous peroxidase activity for DAB staining and IHC, then incubated with 1% BSA in PBS for 1 hour at room temperature to block non-specific binding. Following this, sections were exposed to primary antibodies listed in Supplementary Table S4 overnight at 4°C. After washing, sections were incubated with anti-rabbit IgG as the secondary antibody. DAB staining was used to visualize protein expression, and the reaction was stopped with distilled water. Sections were stained with haematoxylin, dehydrated, and then mounted. Images were captured using an Olympus IX73 fluorescence microscope. In order to perform quantitative analysis of cells, the quantities of SOX9+ cells in every seminiferous tubule of the testis were tallied in five randomly selected fields per section.

To perform immunostaining, the sections underwent permeabilization using 0.1% Tween-20. Following the addition of 3% BSA to obstruct, the sections were subjected to incubation with primary antibodies listed in Supplementary Table S4 overnight at 4°C. On the following day, the samples were exposed to secondary antibodies at ambient temperature in the absence of light for a duration of 1 hour. Additionally, they were stained with Hoechst (Invitrogen, California American) for 10 min at room temperature.

For each group, analysis was conducted on a minimum of three sections obtained from separate experiments. All images were obtained using an IX73 microscope or a Zeiss 700 Laser Scanning Confocal Microscope.

Cell Viability Assays

The cell viability was detected by a CCK-8 kit (Beyotime, Shanghai, China). TM4 cells seeded in 96-well plates at the concentration of 2000 per well were subject to different treatments before adding 10 μL CCK-8 working solution, followed by another 2-hour incubation. A microplate reader Synergy HTX Multi-Mode Microplate Reader (BioTek, Shanghai, China) was used to measure the absorbance at 450nm.

Western blotting

Testes samples and cells were treated and lysed in RIPA buffer supplemented with 1 mM protease inhibitor cocktail and 1 mM phenylmethylsulfonyl fluoride (PMSF) (Beyotime, Shanghai, China). The lysates were sonicated on ice to extract proteins, and after centrifugation at 12,000 r for 1 hour at 4 °C, protein concentrations were determined using the Pierce BCA protein analysis kit (Thermo Fisher Science, MA, USA). Equal amounts of 20 μg protein were separated by 8%-12% SDS–PAGE and transferred to PVDF membranes (Merck Millipore). The membranes were then blocked with 5% BSA for 1 hour and exposed to primary and secondary antibodies successively which were listed in List3. Target protein bands were visualized using the Tanon-5200 Chemiluminescent Imaging System (Tanon Science and Technology) with BlodtightTM Western Chemiluminescent HRP Substrate (Bioworld). The band intensity was further quantified using ImageJ version 1.53t software (National Institutes of Health).

Quantitative RT-PCR

The mRNA expression levels were quantified using quantitative RT-PCR. Total RNA was extracted using a Total RNA Isolation Kit (Cowin Biotech, Jiangsu, China), and 1 μg total RNA was then reverse-transcribed with PrimeScript RT Master Mix (Accurate Biotechnology, Hunan, China). For quantitative RT-PCR, transcripts were amplified using AceQ qPCR SYBR Green Master Mix(Accurate Biotechnology, Hunan, China), and fluorescence signals were monitored by a Roche LightCycler 96 Real-time PCR system (Roche Diagnostics, Basel, Switzerland). The gene gapdh was used as an internal control, and the relative expression levels were figured up using the $2^{-\Delta\Delta Cq}$ method. The sequences of the primers used in this study are listed in Supplementary Table S5.

Detection of Cellular senescence assay

To measure cellular senescence, SA-β-gal activity was assessed with a senescence β-galactosidase staining kit (Beyotime, Shanghai, China). TM4 were seeded in six-well plates, after treated cells were washed 3 times with PBS and fixed with 4% PFA at room temperature for 15 min. After washing with PBS, cells were stained with 1 ml of freshly prepared β-gal detection solution at 37°C overnight. The cells were washed twice with PBS and overlaid with PBS. Images were captured under a microscope and the ratio of blue-stained cells to the total number of cells were indicated for cellular senescence.

Detection of Reactive Oxygen Species (ROS)

The generation of cytosolic ROS was detected using DCFH-DA and Mitochondrial ROS was detected using MitoSOX to indicate mitochondrial superoxide. Briefly, after treatment, TM4 cells were loaded with 10μM DCFH-DA (KeyGEN BioTECH, Nanjing, China) or 5μM MitoSOX (Invitrogen, Thermo Fisher Scientific, Inc.) diluted in DMEM/F12 for 10 min at 37°C. Then the cells were washed twice with PBS, and images were acquired on a fluorescence microscope.

Detection of relative calcium (Ca^{2+}) concentration

Cytosolic Ca^{2+} indicator Fluo-3, mitochondrial Ca^{2+} indicator Rhod-2 and ratio-type dual-wavelength dye Fura-10 were used to stain TM4 cells.

After treatment, TM4 cells were incubated with 2 μM Fluo-3 (KeyGEN BioTECH) or 1 μM Rhod-2 (Abcam) diluted in phenol red-free DMEM/F12 medium for 30 min. Fura-10 AM (AAT Bioquest) was added at a final concentration of 5 μM, and F-127 was added at a final concentration of 0.05%. Then the cells were washed twice with PBS, and images were acquired on a fluorescence microscope. For Fura-10, F340/F380 indicated the baseline calcium levels in treated TM4 cells. Rhod-2 fluorescence intensity was assessed using a versatile microplate reader with excitation/emission wavelengths at Ex/Em=540/590 nm cutoff 570 nm. Additionally, fluctuations or responses in calcium signals are observed following stimulation with ATP (10 μM), which is known to bind IP3R and can enhance Ca^{2+} release, and 2-APB (100 μM), an IP3R inhibitor, to assess the function of calcium channels IP3R.

Detection of Mitochondrial Membrane

Enhanced mitochondrial membrane potential assay kit with JC-1 (Beyotime, Shanghai, China) was used to detect Mitochondrial membrane potential. After treatment, TM4 cells were incubated with JC-1 staining solution at 37 °C for 30 min. After incubation, cells were washed twice with JC-1 buffer at room temperature, then transferred to phenol red-free DMEM/F12 medium. Fluorescence intensity values were detected using a fluorescence microscope and calculated the ratio of JC-1 aggregate form to JC-1 monomer form.

Detection of mitochondrial permeablity transition pore (MPTP)

The mPTP opening of treated TM4 cells was detected using an MPTP Fluorescence Assay Kit (Abbkine, Wuhan, China) according to the manufacturer's instructions. Briefly, after two washes with prewarmed PBS, the cells were treated with Calcein AM staining solution and incubated at 37 °C for an hour in the dark. And then incubation with $CoCl_2$ (1 mM), the green fluorescence of Calcein in the cytoplasm is quenched, leaving only the green fluorescence within the mitochondria. As a positive control, treatment of cells with Ionomycin (0.25 μM) induces a large influx of extracellular Ca^{2+} into the cells and leads to the opening of the MPTP. The cells were washed twice with PBS and observed using a fluorescence microscope. Samples that can induce partial or complete opening of the MPTP will exhibit a decrease or loss of green fluorescence in the mitochondria. By measuring the intensity of green fluorescence in the mitochondria, the opening of the MPTP can be qualitatively and quantitatively assessed.

Mitochondria and endoplasmic reticulum staining and analysis.

The mitochondrial structural network was detected by 200 nM Mito-tracker Red and ER-tracker Green (Beyotime, Shanghai, China) in a culture medium at 37 °C for 30 min. Cells were

imaged using a Zeiss 700 Laser Scanning Confocal Microscope.

Detection of ATP content

A firefly luciferase-based ATP assay kit (Beyotime, Shanghai, China) was used to measure ATP content in the treated TM4 cells, according to the manufacturer's instructions. Briefly, cells were treated and lysed in RIPA buffer supplemented with 1 mM protease inhibitor cocktail and 1 mM phenylmethylsulfonyl fluoride (PMSF). The lysates were sonicated on ice to extract proteins, and after centrifugation at 12,000 r for 1 hour at 4°C, protein concentrations were determined using the Pierce BCA protein analysis kit (Thermo Fisher Science, MA, USA). Equal amounts of protein were mixed with the ATP detection working solution in a white 96-well plate. Standard curves were also generated. Total ATP levels were expressed as nmol/mg protein.
